# Investigation of the mechanism of hypertension caused by BTKi in the treatment of hematologic diseases

**DOI:** 10.3389/fphar.2025.1585061

**Published:** 2025-05-15

**Authors:** Jiayi Xu, Junling Lin, Haojian Gan, Qingjian He, WenJuan Wang, Yuanhua Liu

**Affiliations:** ^1^ Department of Cardiovascular Center, First Affiliated Hospital of Huzhou University, Huzhou, China; ^2^ Department of Breast and Thyroid Surgery, First Affiliated Hospital of Huzhou University, Huzhou, China; ^3^ School of Medicine, Huzhou University, Huzhou, China

**Keywords:** BTKi, ibrutinib, oxidative stress, hypertension, cardiovascular diseases

## Abstract

Bruton’s tyrosine kinase inhibitors (BTKis) have made substantial impacts on the treatment of B-cell malignancies like chronic lymphocytic leukemia (CLL) and small lymphocytic lymphoma (SLL). Therapeutic benefits aside, the clinical use of BTKis comes with several side effects, of which hypertension (HTN) is quite common and serious and of significant clinical concern. The present article will discuss the mechanisms by which the use of BTKis causes hypertension and outline strategies for managing the condition within the clinic. Studies indicate that using BTKis interferes with BTK’s central role within the B-cell receptor (BCR) signaling cascade and impacts multiple downstream signaling pathways like PI3K/Akt, MAPK, and NF-κB. These changes contribute to endothelial dysfunction, increased oxidative stress, and vascular constriction, all of which are implicated in the development of hypertension. Of special concern is that oxidative stress (OS) is directly related to decreased endothelial nitric oxide (NO) production, a finding that becomes particularly relevant during the initiation of BTKi therapy. Also, BTKis affect vascular development and tone regulation by activating the Notch and RhoA/ROCK pathways, leading to increased vasoconstriction and the advancement of hypertension. In light of the possibility that BTKi-induced hypertension might jeopardize treatment tolerability and patient outcomes, this review proposes a multimodal management of the condition, including careful monitoring of blood pressure, individualized antihypertensive treatment, and possible modifications of the dosing of BTKis. Future investigations should look into the specific molecular mechanisms underpinning the development of hypertension due to BTKis as well as the effects of various antihypertensive regimens on the improvement of the cardiovascular profile of affected individuals.

## 1 Introduction

### 1.1 Overview of Bruton’s tyrosine kinase inhibitors (BTKis) in hematologic malignancies and clinical application

The development and application of Bruton’s tyrosine kinase inhibitors (BTKi) have significantly improved the treatment landscape of the B-cell lymphomas (BCL). The drugs have been very effective, particularly in the treatment of chronic lymphocytic leukemia/small lymphocytic lymphoma (CLL/SLL), mantle cell lymphoma (MCL), Waldenström’s macroglobulinemia (WM), marginal zone lymphoma, and diffuse large B-cell lymphoma (DLBCL) (Tam and Thompson, 2024; Hampel and Parikh, 2023).

First-generation Bruton’s tyrosine kinase (BTK) inhibitor ibrutinib was the first of this class of drugs introduced as a central therapeutic agent for chronic lymphocytic leukemia (CLL), ushering in the era of kinase-targeting medications for this disease (Burger and Chiorazzi, 2013). Approval of ibrutinib was prompted by the outcomes of the pivotal 2014 phase 1b/2 trial (PCYC 1102). The prosecution highlighted the drug’s effectiveness among treatment-naive as well as relapsed/refractory (R/R) CLL/SLL participants (Dangi-Garimella, 2014; Byrd et al., 2020a). With a follow-up of 26 months, the approximate 7-year progression-free survival rate (PFS) of treatment-naive cases was 83%, and of R/R was 34%, and the overall response rate (ORR) was 89% (Byrd et al., 2020a; Lipsky and Lamanna, 2020). The second-generation BTKis, acalabrutinib and zanubrutinib, launched in 2017 and 2019, respectively, are a newer class of drugs with lower off-target effects. Both of these drugs are used for CLL, SLL, and MCL (Hata et al., 1998). The first- and second-generation of BTKis are both covalent (irreversible) blockers of BTK. Ibrutinib continues to be the most widely investigated and tracked drug in the clinic as the first-choice treatment in CLL cases (Lipsky and Lamanna, 2020). Aside from the expected inhibition of BTK, ibrutinib also inhibits multiple off-target kinases such as epidermal growth factor receptor (EGFR), ErbB2, interleukin-2-induced T-cell kinase (ITK), and the tyrosine kinase expressed in hepatocellular carcinoma (TEC), thus imparting a profile of toxicities (Jiang et al., 2024). On the other hand, acalabrutinib and zanubrutinib have lower off-target activity, therefore lessening side effects. According to preclinical evidence, second-generation BTKis are less likely to affect tyrosine kinases like EGFR and ITK (Chen et al., 2022). Pirtobrutinib, a new non-covalent (reversible) BTKi, represents the third generation of the BTK inhibitors. It has the advantage of overcoming resistance mediated by the Cys-481 mutation since it does not bind to Cys-481 (Smith and Burger, 2021). Furthermore, pirtobrutinib is the first BTKi demonstrated to have sustained efficacy among heavily pretreated R/R MCL patients. Better tolerability, decreased toxicity, and lower discontinuation rates are evident for pirtobrutinib over first- and second-generation BTKis, although more clinical studies and data are required to determine its long-term results and safety (Mato et al., 2021). It should also be noted that if the tumor is not dependent on the B-cell receptor (BCR) signal, the BTKi treatment would be ineffectual (Wang et al., 2021).

### 1.2 Adverse effects of BTKis, with a focus on hypertension

Bruton’s tyrosine kinase inhibitors (BTKis) have proven more effective than traditional chemotherapy and immunotherapy in treating a variety of hematologic malignancies, with a generally favorable safety profile. However, accumulating data indicate the presence of several adverse events, particularly cardiovascular disorders. In one study, the median treatment duration for acalabrutinib was 38.3 months (range: 0.3–55.9 months), while for ibrutinib it was 35.5 months (range: 0.2–57.7 months). Among the most common adverse events (AEs) of any severity, occurring in at least 10% of patients in both groups, were diarrhea, headache, cough, joint pain, bruising, atrial fibrillation, hypertension, urinary tract infections, back pain, muscle cramps, and dyspepsia (Byrd et al., 2021; Wiczer et al., 2017). A retrospective cohort study focusing on chronic lymphocytic leukemia (CLL) patients identified atrial fibrillation as the primary reason for discontinuing ibrutinib. This condition is associated with a heightened risk of both overall mortality and cardiovascular deaths, including stroke and other cardiac complications. Managing atrial fibrillation is particularly challenging, as the concurrent use of anticoagulants with BTK inhibitors increases the risk of bleeding (Chai et al., 2017). Long-term follow-up data from a phase II clinical trial on acalabrutinib monotherapy in previously untreated or relapsed/refractory CLL patients showed that 6% and 11% of patients, respectively, discontinued treatment due to adverse events, following median follow-up periods of 53 and 41 months (Byrd et al., 2020b). These adverse reactions are attributed to the inhibition of BTK by BTK inhibitors and the variable off-target effects on other kinases such as interleukin–2–inducible T-cell kinase (ITK), tyrosine kinase (TEC), and epidermal growth factor receptor (EGFR). The toxicities observed are closely related to the binding patterns of these drugs with their target kinases (Lipsky and Lamanna, 2020).

There is growing interest in understanding the long-term effects of hypertension, particularly hypertension associated with cancer treatment, on patient morbidity and mortality (GBD, 2017 Causes of Death Collaborators, 2018). Hypertension is the most common modifiable risk factor for cardiovascular disease and persists as a significant issue following acute cancer treatment, affecting both adult and pediatric cancer survivors (GBD, 2017 Causes of Death Collaborators, 2018). The incidence of hypertension in cancer survivors is 2.5 times higher than in the general population, further increasing the cardiovascular disease risk and mortality already elevated in these patients (Armenian et al., 2016). A retrospective analysis of 562 patients treated with ibrutinib revealed that 78% of patients developed new or worsened hypertension, with half of these events occurring within 2 months of initiating ibrutinib therapy (Ahn and Brown, 2021). Despite this, research and clinical data on hypertension induced by BTK inhibitors (BTKis) remain limited. Much of the data on cancer treatment-related hypertension stems from oncology trials primarily focused on assessing anticancer drug efficacy and cancer prognosis, with limited cardiovascular data collected. This clearly emphasizes the need for larger, higher-quality studies focusing on specific cardiovascular outcomes (Armenian et al., 2016; Gibson et al., 2017). There is an urgent need for dedicated trials on blood pressure management in cancer patients and survivors, aiming to study the incidence and mechanisms of hypertension following BTKi treatment (Chow et al., 2014; Sturgeon et al., 2019).

### 1.3 Rationale for investigating BTKi-induced hypertension

The potential mechanisms behind BTKi-induced hypertension are multifactorial. Evidence suggests that BTK inhibition leads to an imbalance between the oxidative and antioxidant systems, resulting in endothelial dysfunction that reduces vascular tone and enhances contraction (Gallo and Savoia, 2024). The PI3K/Akt, MAPK, and NF-κB signaling pathways, which are located downstream of the B-cell receptor (BCR) signaling pathway, interact intricately with BTK, a pivotal kinase in this network (EB Gomez et al., 2023). After BTKi administration, BTK inhibition may decrease activity in these downstream pathways, including PI3K/Akt, MAPK, and NF-κB. Another possibility is that, beyond inhibiting BTK, BTKis may interact with other kinases sharing similar structural domains, thereby blocking these pathways. These signaling pathways are each crucial in regulating vascular blood pressure through distinct mechanisms (T Zou et al., 2010; TM Seccia et al., 2020; Slowinski et al., 2007). In addition, two other pathways, the Notch and RhoA/ROCK signaling pathways, which regulate endothelial proliferation and vascular tone, have shown potential links to the BTK pathway and may contribute to the hypertension induced by BTKis (Zhou et al., 2022; Fulton et al., 1999).

In conclusion, the pathophysiology of BTKi-induced hypertension is driven by the complex interactions between oxidative stress, endothelial dysfunction, and alterations in signaling pathways. Further investigation is needed to uncover the precise molecular mechanisms involved and to identify possible therapeutic strategies to mitigate this adverse effect. Gaining insights into these mechanisms will be crucial for formulating targeted interventions to manage hypertension in patients treated with BTKi, ultimately enhancing both cardiovascular health and cancer outcomes.

## 2 BTK and the mechanism of action of BTKis

### 2.1 Role of BTK in B-cell receptor (BCR) signaling

The B-cell receptor (BCR) signaling pathway plays a vital role in B-cell development and contributes to the pathogenesis of B-cell tumors (Ahn and Brown, 2021). Bruton’s tyrosine kinase (BTK) is a multi-domain protein containing SRC homology 2 (SH2) and SH3 domains, an amino-terminal pleckstrin homology (PH) domain, a proline-rich TEC homology (TH) domain, and a catalytic domain. It serves as a fundamental component of the B-cell receptor signaling complex (Tinworth et al., 2019). BTK activation occurs in two steps: (1) Phosphorylation of BTK at the Y551 site in the kinase domain by spleen tyrosine kinase (SYK) or SRC family kinases; (2) Phosphorylation at Y551 results in autophosphorylation at the Y223 site in the SH3 domain, which fully activates BTK’s kinase activity and stabilizes its active conformation (Pal et al., 2018). The phenotype of X-linked agammaglobulinemia (XLA) patients and the finding that BTK inhibition blocks downstream signaling underscores BTK’s central role in the BCR signaling pathway (Honigberg et al., 2010). As a critical protein in BCR signal transduction, BTK is an attractive drug target for B-cell malignancies (such as chronic lymphocytic leukemia and mantle cell lymphoma) and autoimmune and inflammatory disorders (Mouhssine et al., 2024). In B-cell malignancies, BCR signaling is sustained through ligand and ligand-independent mechanisms, resulting in continuous BTK activation (Tkachenko et al., 2023). This persistent activation provides a survival and proliferative advantage to tumor clones in B-cell malignancies (Maher et al., 2023).

### 2.2 Mechanism of BTK inhibition by covalent/reversible binding to Cys-481 in the ATP-binding domain

Based on their mechanisms of action and binding modes, BTK inhibitors (BTKis) are divided into two types: (i) covalent/irreversible inhibitors, such as the first-generation BTKi ibrutinib and the second-generation BTKi acalabrutinib. These inhibitors are characterized by a Michael receptor fragment that forms a permanent covalent bond to the conserved Cys481 residue located at the ATP-binding site. This interaction effectively occupies the binding site, thereby preventing the phosphorylation of downstream targets such as Akt and PLC-γ2 (Maher et al., 2023). Consequently, BTK signaling is disrupted, leading to inhibition of the BCR pathway both *in vitro* and *in vivo* (Mouhssine et al., 2024). (ii) Non-covalent/reversible inhibitors, which bind to a specific pocket in the SH3 domain through weak, reversible interactions (such as hydrogen bonds or hydrophobic forces), causing the enzyme to adopt an inactive conformation (Tinworth et al., 2019). Recently, a novel class of hybrid BTK inhibitors has been introduced. These inhibitors bind to BTK through a reversible covalent interaction, forming a temporary covalent bond with the Cys481 residue. This mechanism results in the transient inactivation of the enzyme (Tasso et al., 2021). These inhibitors are distinguished by their high potency and selectivity, extended and adjustable residence times, and fewer off-target effects, merging the advantages of both covalent and non-covalent inhibitors (Zhao et al., 2021). Covalent BTK inhibitors, such as ibrutinib and acalabrutinib, leverage the presence of the Cys481 residue in BTK to achieve strong binding affinity, resulting in effective BTK inhibition. However, the selectivity of these inhibitors is limited. In addition to their intended effects, ibrutinib also induces off-target inhibition of several non-target proteins, including EGFR, ErbB2, ITK, and TEC (Ganatra et al., 2018; Kaur and Swami, 2017). Although these off-target effects contribute to the antitumor efficacy of BTK inhibitors, they are also associated with a range of adverse events, including hypertension, atrial fibrillation, bleeding complications, and impaired macrophage phagocytosis (Gabizon and London, 2020; Borge et al., 2015). Additionally, resistance to BTK inhibitors has been linked to mutations at the Cys481 residue, which prevent the covalent binding of ibrutinib, acalabrutinib, and zanubrutinib. As a result, these inhibitors only achieve temporary inhibition of the mutated BTK protein (Maher et al., 2023). The advent of non-covalent BTK inhibitors, such as pirtobrutinib, provides a theoretical means to overcome resistance driven by these mutations. Additionally, targeting BTK using proteolysis-targeting chimeras (PROTACs) emerges as a promising strategy to address BTKi resistance in B-cell malignancies (Mouhssine et al., 2024).

### 2.3 Downstream effects on BCR-related pathways

**FIGURE 1:**
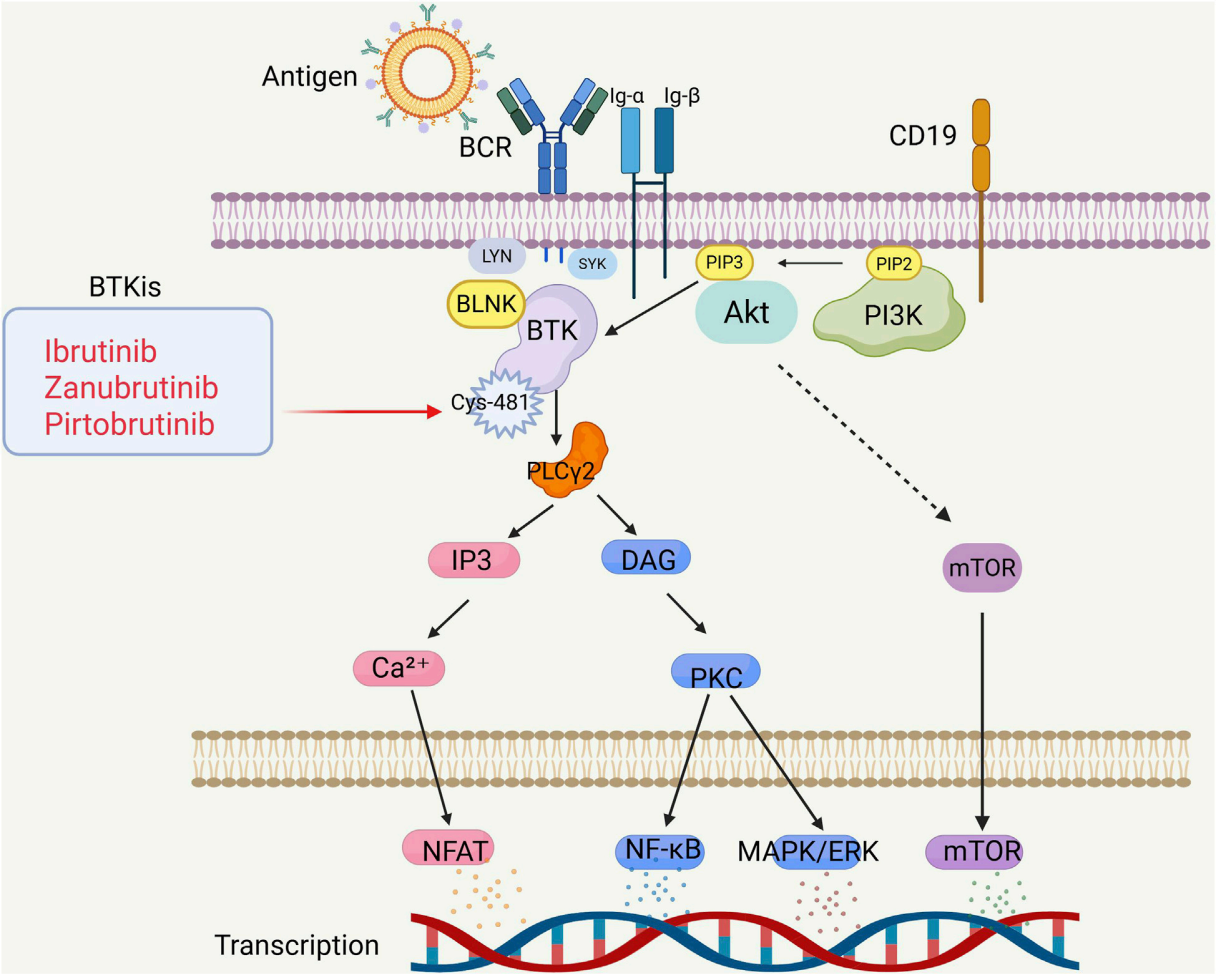
BCR signaling pathway and mechanism of action of BTKi. B-cell receptors consist of transmembrane immunoglobulin (Igα/β) and CD19, which are responsible for antigen recognition and activation of downstream signaling cascades. IP3-Ca^2+^-NFAT pathway: promotes Ca^2+^ release, activates NFAT (nuclear factor-activated T cells), and regulates gene transcription. DAG-PKC pathway: activation of PKC (protein kinase C), further activation of NF-κB and MAPK/ERK, regulation of inflammatory response and cell survival. PI3K-AKT-mTOR pathway: promotes B cell survival and metabolic regulation through AKT and mTOR. BTKi inhibits BTK activity by covalently or non-covalently binding to Cys-481 (cysteine-481), blocking BCR signaling and inhibiting B cell survival. Image created with BioRender.com (accessed on 9 May 2025).

BCR signaling is interconnected through a network of kinases and phosphatases, which regulate and amplify its activation. In general, BCR signaling is categorized into chronic activation BCR and rigid BCR (Profitós-Pelejà et al., 2022). Chronic activation BCR is an antigen-dependent process, primarily engaging pathways like NF-κB and MAPK/ERK. In contrast, rigid BCR sustains B-cell survival *via* a non-antigen process, primarily through the PI3K/Akt pathway (Lam et al., 1997). When antigens bind to immunoglobulins on the B-cell surface, BCR signaling is initiated, leading to the coupling and autophosphorylation of immunoreceptor tyrosine-based activation motifs (ITAMs) on the cytoplasmic tails of CD79A (Igα) and CD79B (Igβ), a process facilitated by the protein kinase LYN, a member of the Src family kinases (Ahn and Brown, 2021). Phosphorylation triggers the recruitment of various signaling complexes, such as SYK, Bruton’s tyrosine kinase (BTK), phospholipase Cγ2 (PLCγ2), and protein kinase C (PKC) (Woyach et al., 2012). BTK activation is initiated when the Src homology 2 (SH2) domain of the adaptor protein BLNK is recruited, making BLNK a key component of the signaling complex associated with the activated CD79 complex (Wang et al., 2015). Once bound, BTK is phosphorylated by SYK. BLNK functions as a scaffold, enabling the binding of PLC-γ2, which is then phosphorylated by both SYK and BTK (Pal et al., 2018). Additionally, LYN can directly phosphorylate PLC-γ2 (Kabak et al., 2002). Active PLC-γ2 hydrolyzes phosphatidylinositol (PIP2), generating the second messengers diacylglycerol (DAG) and inositol trisphosphate (IP3). IP3 stimulates calcium influx, which activates PKC β (Engels et al., 2001). The calcium influx then activates calmodulin-dependent phosphatase, leading to the phosphorylation of NFAT (Rao et al., 1997). PKC β directly phosphorylates various mitogen-activated protein transcription factors, including ERK, JNK, and p38/MAPK, which activate the mitogen-activated protein kinase (MAPK) pathway (Jacob et al., 2002). NF-κB is indirectly activated through the CARD11-BCL-10-MALT1 complex, leading to the phosphorylation of IκB, which releases NF-κB and allows its translocation to the nucleus (Stadanlick et al., 2008). Activation of BCR also results in the recruitment of CD19, which is phosphorylated by LYN. This phosphorylation facilitates the binding of the p85 subunit of PI3K to CD19, thereby activating the p110 δ subunit in B cells. PI3K then phosphorylates PIP2, generating phosphatidylinositol 3,4,5-trisphosphate (PIP3) (Burger, 2019). PIP3 is essential for recruiting BTK and activating PLC-γ2, thereby linking PI3K signaling to calcium flux and the subsequent activation of Akt and mTOR (Patton and Woyach, 2024). (Figure 1).

## 3 BTK inhibition and blood pressure regulation

### 3.1 Clinical observations and prevalence in patients undergoing BTKi therapy

In clinical settings, some patients receiving BTKi treatment experience varying levels of hypertension. A phase III open-label, randomized controlled trial with a median follow-up of 44.4 months showed that the incidence of hypertension in Waldenström’s macroglobulinemia (WM) patients after treatment with ibrutinib and zanubrutinib was 25.5% and 14.9%, respectively (Dimopoulos et al., 2023). A study by Dickerson et al., which examined over 500 cancer patients who received ibrutinib treatment between 2009 and 2016, found that more than three-quarters of patients developed new or aggravated hypertension within 30 months of treatment (Dickerson et al., 2019). A statistical analysis of a long-term follow-up study of up to 6 years in patients with chronic lymphocytic leukemia (CLL) or small lymphocytic lymphoma treated with ibrutinib found that 21% of patients developed hypertension (Munir et al., 2019). In a randomized controlled clinical trial with a median follow-up of 42.5 months, the incidence of hypertension in 325 and 327 patients with relapsed/refractory CLL and small lymphocytic lymphoma after treatment with ibrutinib and zanubrutinib was 25.3% and 27.2%, respectively (Brown et al., 2024). Moreover, in a clinical trial of 300 CLL patients treated with ibrutinib, new-onset hypertension occurred in 68.5% of patients (I Goh and Chew, 2018). Similar results were observed in an open-label phase III clinical trial (Barr et al., 2018). Additionally, clinical trials conducted by Tam C, Moslehi JJ, Ryan CE, and others confirmed that hematologic cancer patients undergoing prolonged BTKi therapy experience varying degrees of hypertension (Tam and Thompson, 2024; Moslehi et al., 2024; Ryan et al., 2023). A pooled analysis of 424 patients from three phase III trials of ibrutinib in chronic lymphocytic leukemia (CLL) revealed that 18% of patients developed hypertension, with 6% experiencing severe hypertension after a period of treatment (Abdel-Qadir et al., 2017). A study with a median follow-up of 41 months found that 59.2% of patients developed new or worsened hypertension, with 35% showing an increase in systolic blood pressure of ≥10 mmHg from baseline and 13.5% showing an increase of ≥20 mmHg. Among patients without baseline hypertension, 62 (53.9%) developed new hypertension after starting acalabrutinib treatment, with an average increase in systolic blood pressure (SD) of 16.7 mmHg. The median time to reach the maximum increase in systolic blood pressure was 15 months (Chen et al., 2022). These findings indicate a high incidence of hypertension following BTKi therapy, and the emergence of malignant hypertension or hypertension-related complications often results in the discontinuation of treatment. This emphasizes the need for vigilant monitoring of hypertension during BTKi therapy and calls for further research to reduce its occurrence or prevent its onset (Table 1).

**TABLE 1:** Incidence of hypertension during BTKi treatment.

BTKI	Author	Incidence of hypertension	Type of study
Ibrutinib	Dimopoulos MA	25.5%	Phase III randomized controlled trial
Zanubrutinib	Dimopoulos MA	14.9%	Phase III randomized controlled trial
Ibrutinib	TalhaMunir	21%	RESONATE randomized controlled clinical trial
Ibrutinib	Jennifer R Brown	25.3%	ALPINE Phase III randomized controlled trial
Zanubrutinib	Jennifer R Brown	27.2%	ALPINE Phase III randomized controlled trial
Ibrutinib	Gordon MJ	68.5%	Retrospective Cohort Study
Acalabrutinib	Dickerson T	24.4%	Meta-Analysis
Acalabrutinib	Dickerson T	20.5%	Meta-Analysis
Ibrutinib	Dickerson	75.0%	Meta-Analysis
Acalabrutinib	Sunnia T	48.9%	Retrospective cohort study
Ibrutinib	Laura Samples	39.8%	Multicenter retrospective study
Ibrutinib	Robert A Redd	40.0%	Retrospective cohort study
Ibrutinib	Srilakshmi Vallabhaneni	6.3%	Retrospective cohort study
Acalabrutinib	Jérôme Paillassa	32.0%	Retrospective cohort study

### 3.2 Impact on cardiovascular complications and prognosis

Due to the confounding factors associated with elevated blood pressure in cancer patients, particularly during times of heightened stress, potential fear of negative news, and the critical focus on managing life-threatening diseases, increased blood pressure may not consistently be recognized in clinical care settings. However, it has been noted that in patients undergoing BTKi treatment, the progression of hypertension can lead to an unexpected increase in the burden of cardiovascular events (Chen et al., 2022). Currently, there is limited research and data on the relationship between BTKi-induced hypertension and Major Adverse Cardiovascular Events (MACE), as well as its long-term impact on cardiovascular complications. In a clinical study with a median follow-up of 41 months, 41 patients (14.6%) experienced MACE after treatment with acalabrutinib, with 18.2% of these events linked to newly developed or worsened hypertension. In contrast, patients who did not experience new or worsened hypertension after acalabrutinib initiation had an MACE incidence of 11.2%. This suggests that patients with newly developed or worsened hypertension have a higher risk of MACE (18.2% vs 11.2% in those without hypertension progression). However, the difference was not statistically significant following multivariate analysis (Chen et al., 2022).

A retrospective cohort study of lymphoma patients treated at Ohio State University Cancer Center revealed that 93 patients (16.5%) experienced MACE, with 84 of them (19.1%) having new or worsened hypertension, and nine patients (8.2%) remaining stable or without hypertension. Multivariate regression analysis showed that new or worsened hypertension was associated with an increased risk of MACE. Atrial fibrillation was the most common cardiovascular complication during ibrutinib therapy, occurring in 73 patients (13%), followed by new-onset heart failure (3.7%), cerebrovascular events (2.1%), myocardial infarction (1.4%), and ventricular arrhythmias or sudden cardiac death (1.1%) (Dickerson et al., 2019). In the first year of acalabrutinib treatment, the extent of early systolic blood pressure elevation was associated with the subsequent risk of atrial fibrillation (AF). For every 5 mmHg increase in systolic blood pressure, the risk of MACE increased by 27% (0.27) (P < 0.001), and the risk of AF development increased by 42%. Interestingly, in another study, baseline hypertension was not associated with major MACE, which was defined as new-onset coronary artery disease, congestive heart failure, atrial fibrillation, stroke, or cardiovascular death. This finding contrasts with the results of the previous two studies, possibly due to differences in follow-up durations. The Chen ST and Dickerson studies had follow-up periods of 41 months and 30 months, respectively, whereas this study had a 5-year follow-up (I Goh and Chew, 2018).

Moreover, managing hypertension during BTKi treatment often requires combination therapy. The most frequently used treatments for hypertension were diuretics (22.4%), followed by angiotensin blockers (18%), receptor blockers (15.4%), calcium channel blockers (12.4%), and other treatments (3.6%; e.g., clonidine). In the monotherapy group, there were no significant differences in long-term systolic blood pressure (SBP) control across different antihypertensive drug classes. In the combination therapy group (≥2 drugs), there was a trend toward SBP reduction, but it did not reach statistical significance. Among baseline monotherapy patients (21.9%), no antihypertensive class significantly prevented the progression of hypertension (Dickerson et al., 2019). When BTKi-associated hypertension develops and remains uncontrolled despite the use of multiple antihypertensive drugs, it is reasonable to discuss with oncologists and patients the possibility of reducing chemotherapy doses or implementing chemotherapy breaks. It may also be prudent to contemplate reducing the dose or temporarily discontinuing other medications that could exacerbate hypertension, including nonsteroidal anti-inflammatory drugs (NSAIDs), erythropoiesis-stimulating agents, and high-dose corticosteroids (Cohen et al., 2019a). Managing hypertension can reduce the long-term risk of subsequent MACE events, so aggressive antihypertensive treatment should be initiated when hypertension develops or worsens. However, additional prospective studies are required to validate these findings.

## 4 Oxidative stress and BTKi-Induced hypertension

### 4.1 Oxidative stress as a key contributor to endothelial dysfunction and vascular tone

Oxidative stress (OS) occurs when the excessive production of reactive oxygen species (ROS) surpasses the buffering capacity of the antioxidant defense system, or when there is a deficiency in antioxidant enzymes (Uttara et al., 2009). ROS are active intermediates of molecular oxygen and act as important intracellular second messengers. In vascular tissues, enzymes that produce ROS include NADPH oxidase (NOX), xanthine oxidase, and the mitochondrial respiratory chain, and their increased activity can lead to higher ROS levels in the vasculature (Citrin et al., 2024). ROS consist of free radicals (such as superoxide anion (⋅O2), hydroxyl radical (⋅HO), and nitric oxide (⋅NO)) and non-radical oxygen species (such as H2O2, HOCl, and ONOO−) (Watson et al., 2008). Superoxide anion can directly react with NO, leading to the generation of peroxynitrite, which has been shown to uncouple endothelial nitric oxide synthase (eNOS), resulting in decreased NO synthesis (Mollnau et al., 2002). Under normal physiological conditions, there is a balance between the production of reactive oxygen species (ROS) and the clearance of toxic compounds in the endothelium through endogenous antioxidants (Gutteridge and Mitchell, 1999). However, under certain pathological and physiological conditions, such as hyperlipidemia, ischemia-reperfusion injury, and shear stress damage, this balance between ROS production and antioxidant defense mechanisms can be disrupted. This disruption leads to oxidative stress, which can induce endothelial cell dysfunction through various pathways, thereby exacerbating the progression of cardiovascular diseases (Zhang et al., 2023).

The endothelium is a single-cell layer that lines the inner surface of blood vessels. It plays a crucial role in maintaining vascular homeostasis, regulating vascular tone and permeability, and carrying out anti-inflammatory, antioxidant, anti-proliferative, and anti-thrombotic functions (Gallo and Savoia, 2024).

Endothelial dysfunction (ED) can contribute to a range of diseases, such as atherosclerosis, diabetes, coronary artery disease, hypertension, and hypercholesterolemia (Incalza et al., 2018). It is characterized by an imbalance between vasodilators and vasoconstrictors, which promotes conditions favorable to thrombosis and an atherosclerotic phenotype. This dysfunction manifests in various ways, including vasoconstriction, leukocyte adhesion, platelet activation, vascular inflammation, impaired coagulation, increased oxidative stress, and the development of atherosclerosis and thrombosis (Dhananjayan et al., 2016; Verma and Anderson, 2002).

Nitric oxide (NO), the primary vasodilator produced by endothelial cells, is mainly synthesized by endothelial nitric oxide synthase (eNOS) through the conversion of L-arginine to L-citrulline (Holland et al., 1998). NO regulates vascular dilation and constriction by interacting with smooth muscle cells (SMCs) (Hsich et al., 2000). This process involves the activation of soluble guanylate cyclase (sGC) and protein kinase G (PKG), leading to an increase in cyclic guanosine monophosphate (cGMP) levels and ultimately causing vasodilation (Imanishi et al., 2005). Additionally, NO plays an essential role in inhibiting SMC proliferation, inflammation, and platelet aggregation, contributing to the overall maintenance of vascular function. A reduction in NO bioavailability is a critical factor in endothelial dysfunction in patients with cardiovascular disease (Imanishi et al., 2005). Oxidative stress plays a significant role in regulating eNOS activity and NO bioavailability. Elevated intracellular reactive oxygen species (ROS) levels can reduce eNOS activity by decreasing BH4 or L-arginine levels, leading to eNOS uncoupling and producing superoxide rather than NO (Meza et al., 2019). Furthermore, high ROS levels can chemically inactivate bioactive NO by forming peroxynitrite, worsening oxidative stress, and decreasing NO’s effectiveness as a vasodilator through eNOS uncoupling (Li H. et al., 2019). The decline in NO bioavailability marks the onset of endothelial dysfunction. Additionally, ROS affects low-density lipoprotein (LDL), and once oxidized, LDL can inactivate NO and its production, leading to endothelial dysfunction (Griendling et al., 2000). Excessive oxidative stress can also cause mitochondrial dysfunction, enzyme deficiencies, and/or uncoupling, further increasing oxidative stress and enhancing inflammatory responses. Oxidative stress also promotes the EndMT process and inhibits endothelial vasculogenesis (Zhang et al., 2023). As such, oxidative stress plays a central role in developing endothelial dysfunction.

### 4.2 BTK’s role in NADPH oxidase regulation (potential link to NOX2 and ROS production)

The NADPH oxidase complex consists of transmembrane components (gp91phox and p22phox), cytosolic components (p47phox, p67phox, and p40phox), and Rac2 (Sumimoto, 2008). The NOX enzyme family includes various members, such as NOX1, NOX2, NOX3, NOX4, and NOX5 (Pagano et al., 1997). NOX2 is primarily found in neutrophils and macrophages, which play a major role in ROS production in immune cells. Excessive activation of NOX has been identified as one of the most important mechanisms of reactive oxygen species generation (Violi et al., 2020). Although initially described in phagocytic immune cells (like neutrophils), the NADPH oxidase system is now known to be present in most types of vascular cells, including endothelial cells and smooth muscle cells (Griendling et al., 2000). By generating ROS, NADPH oxidase profoundly influences endothelial cell function, vascular tone, and vascular remodeling (Violi et al., 2020). Inhibitors of NOX may provide a promising approach to treating vascular diseases related to oxidative stress (Jaquet et al., 2009).

Currently, there is limited research directly linking BTK with NADPH oxidase, though some studies suggest that BTK regulates NADPH oxidase activity. The precise interaction between these two remains unclear. Honda F and colleagues found that in the absence of BTK, neutrophils produce excessive ROS after stimulation by Toll-like receptors (TLR) or TNF receptors. ROS production is closely tied to NADPH oxidase activation. Specifically, the literature points out that neutrophils lacking BTK exhibit excessive NADPH oxidase activity, leading to overactivation of the cells and initiating apoptosis. BTK regulates NADPH oxidase activation by interacting with Mal, a TLR adaptor molecule. In the absence of BTK, Mal is incorrectly translocated to the cell membrane, where it activates PI3K and enhances NADPH oxidase activity (Honda et al., 2012). However, other studies have shown that BTK enhances neutrophil oxidative bursts (ROS production) and degranulation by activating the p40phox subunit of the NADPH oxidase complex and the small GTPase RAC2, which is crucial for effectively disrupting fungal hyphae. The study also suggests that BTK inhibitors (BTKis) can selectively suppress neutrophil oxidative bursts, degranulation, and the destruction of Aspergillus hyphae, leaving spore phagocytosis and intracellular killing functions unaffected. These findings indicate that BTK regulates NADPH oxidase activity, specifically in neutrophil oxidative responses during antifungal activity (Desai et al., 2024). Research on BTK’s role in acute kidney injury indicates that BTK mediates an increase in renal ROS, contributing to elevated biochemical markers of acute kidney injury, such as serum creatinine/urea nitrogen levels, increased renal medullary peroxidase activity, and histopathological damage to renal tubules. However, BTKi treatment has been shown to reduce oxidative stress in neutrophils, B cells, and kidney tissues, while improving sepsis-induced renal dysfunction (Nadeem et al., 2021). These findings appear to be contradictory, highlighting the need for further research to explore the relationship between BTK, NADPH oxidase, and oxidative stress.

### 4.3 Impact on endothelial function and blood vessel constriction

Considering BTK’s regulation of NADPH oxidase, the significant role of NADPH oxidase in oxidative stress, and the effects of oxidative stress on endothelial dysfunction and vascular tone, it can be concluded that oxidative stress is one of the mechanisms by which BTK inhibitors (BTKis) cause elevated blood pressure. By inhibiting BTK, BTKis may lead to enhanced NADPH oxidase activity, disrupting the balance between oxidative and antioxidant systems within the body. This disruption results in oxidative stress, which reduces NO bioavailability, leading to endothelial dysfunction and vasoconstriction, ultimately contributing to hypertension (Zhang et al., 2023; Griendling et al., 2000; Honda et al., 2012). Therefore, although BTKis are effective in treating certain immune diseases and B-cell tumors, they may have adverse effects on vascular health by the mechanisms outlined above, potentially leading to or worsening hypertension (Samples et al., 2024; Morris G. et al., 2019)。

## 5 Key signaling pathways involved in BTKi-associated hypertension

### 5.1 PI3K/Akt signaling pathway

#### 5.1.1 PI3K/Akt pathway and the ET-1/NO system

PI3K is composed of a catalytic subunit (p110) and a regulatory subunit (p85), and it is typically activated by cell surface receptors, such as receptor tyrosine kinases (RTKs) (Morello et al., 2009). The PI3K family plays a crucial role in regulating several fundamental cellular functions, such as growth, proliferation, metabolism, migration, and secretion (Manning and Toker, 2017). Disruptions in PI3K signaling are associated with a wide range of human diseases, including various cancers, immune system disorders, neurological conditions, diabetes, abnormal tissue growth, and cardiovascular diseases (Li J. et al., 1997). Akt, or protein kinase B (PKB), is a serine/threonine kinase pivotal in cellular signal transduction pathways. Akt is more extensively activated downstream of receptor-mediated PI3K activation than other effectors (Fruman et al., 2017). Upon PI3K activation, PIP3 is generated and forms a signaling platform at the cell membrane. PIP3 recruits Akt and PDK1, leading to Akt phosphorylation and activation. Once activated, Akt regulates various biological processes, such as cell survival, proliferation, and metabolism, by phosphorylating downstream targets. This process is a pivotal regulatory mechanism in numerous physiological processes (such as immune responses and cell growth) and pathological conditions (such as cancer and diabetes) (Vanhaesebroeck et al., 2012).

NO is produced by NOS through an NADPH-dependent process (Engineer et al., 2019). The NOS family consists of three isoforms: neuronal NOS (nNOS), inducible NOS (iNOS), and endothelial NOS (eNOS) (Xu et al., 1999). nNOS is predominantly expressed in the sarcoplasmic reticulum (SR) of cardiomyocytes, while iNOS is mainly expressed in endothelial cells (ECs), vascular smooth muscle cells (VSMCs), cardiomyocytes, neurons, and fibroblasts (Pautz et al., 2010). eNOS is primarily expressed in endothelial cells and cardiomyocytes (Menshikova et al., 2000). NOS activity can be phosphorylated and activated by the PI3K/Akt pathway (Farah et al., 2018). Nitric oxide plays a pivotal role in maintaining cardiovascular homeostasis, regulating vascular tone, and inducing endothelial-dependent vasodilation (Godo and Shimokawa, 2017). Additionally, endothelial-derived NO exhibits antioxidant, antiproliferative, antithrombotic, and anti-inflammatory effects (Vanhoutte et al., 2017).

ET-1 is formed from proendothelin-1 through the action of endothelin-converting enzyme (ECE) (Aflyatumova et al., 2018). Its effects are mainly mediated by ETA and ETB endothelin receptors, which play significant roles in the kidney, lungs, coronary arteries, and cerebral circulation. These receptors are involved in vasoconstriction, pro-inflammatory effects, mitogenesis, cell proliferation, free radical production, and platelet activation (Böhm and Pernow, 2007; Mazzuca and Khalil, 2012). Under normal physiological conditions, ET-1 and NO maintain a delicate balance through complex interactions to support normal vascular function. Furthermore, there exists a complex interplay between the PI3K/AKT signaling pathway and the ET-1/NO system, which will be further explored in the subsequent sections.

#### 5.1.2 BTK inhibition’s effect on PI3K/Akt signaling

PI3K is an essential component of the BCR signaling pathway, playing a critical role in B cell survival, proliferation, and differentiation (Werner et al., 2010). The exact mechanism by which BTKi induces hypertension is still unclear, but inhibition of the PI3K/Akt pathway is a commonly discussed possibility (Lipsky and Lamanna, 2020; Byrd et al., 2021). When the BCR receptor binds to an antigen, phosphorylation of the Igα/Igβ complex recruits the regulatory subunit p85 and the catalytic subunit p110 of PI3K, activating its catalytic activity and converting PIP2 to PIP3 (Lüscher et al., 1990). PIP3 acts as a second messenger, triggering a variety of downstream signaling pathways (Lüscher et al., 1990). BTK is a key kinase in the BCR signaling pathway, and there is a strong relationship between PI3K and BTK functions, with both being integral parts of the BCR signaling network in B cells (Cantley and Songyang, 1994; Fruman et al., 2000). Studies show that BTK interacts with PIP5K (phosphatidylinositol-4-phosphate 5-kinase) to promote the synthesis of PtdIns-4,5-P2, which is a substrate for PI3K and essential for cell signaling (Saito et al., 2003). In the BCR signaling pathway, activation of SYK and BTK mediates the tyrosine phosphorylation of the B cell adapter protein (BCAP), leading to the recruitment of PI3K (Okada et al., 2000). Additionally, BTK binds to PIP3 generated by PI3K *via* its PH domain, which is activated at the cell membrane and contributes to maintaining normal B cell function (Lien et al., 2017). By inhibiting BTK, BTKi disrupts the PI3K/Akt signaling pathway in B cells, resulting in physiological effects. Research has demonstrated that ibrutinib inhibits the PI3K-Akt pathway in the heart, thereby increasing the risk of atrial fibrillation during treatment (McMullen et al., 2014).

#### 5.1.3 Disruption of ET-1 (Endothelin-1) and NO (nitric oxide) balance

ET-1 is a potent vasoconstrictor released by endothelial cells, while nitric oxide (NO) is the primary vasodilator produced by the endothelium, with opposing actions in the vasculature. ET-1 induces vasoconstriction by acting on vascular smooth muscle cells, increasing vascular resistance, while NO relaxes vascular smooth muscle, leading to vasodilation and a reduction in resistance (Rosanò et al., 2013). As mentioned earlier, ET-1 interacts with two types of receptors, ETAR and ETBR (Davenport and Battistini, 2002). ETAR is primarily located on the surface of vascular smooth muscle cells, while ETBR is mainly found on endothelial cells (Kuo et al., 2012). ET-1 binding to ETAR on vascular smooth muscle cells causes vasoconstriction and vasospasm, which worsens ischemia. In contrast, ET-1 binding to ETBR induces the production of NO and prostaglandins, resulting in vasodilation and improved ischemia (Böhm and Pernow, 2007). The interaction between the PI3K/Akt signaling pathway and the ET-1/NO system is intricate and complex. Endothelial ETB receptors mediate the release of ET-1 from the vascular endothelium, and NO released from the endothelium inhibits ET-1-induced vasoconstriction mediated by ETA and/or ETB receptors while also suppressing ET-1 formation and release (Rapoport, 2014). Li L and colleagues found that ETAR siRNA specifically inhibits the expression of ETAR, preventing ET-1 from binding to ETAR. In the absence of ETAR, ET-1 binds more to ETBR, activating PI3K/Akt, which leads to increased NO production. Their study showed that ETAR siRNA primarily enhances NO production *via* the PI3K/Akt pathway through eNOS activation. Additionally, NO production regulates the sGC/cGMP/PKG signaling pathway, modulating the activity of ET-1-related transcription factors (Li L. et al., 2019). Under normal conditions, the balance between ET-1 and NO is vital for maintaining vascular tone and blood pressure stability. Disruptions in this balance (e.g., insufficient NO or excessive ET-1) can cause vasoconstriction and increase vascular resistance, potentially leading to hypertension and related diseases. Chronic ET-1/NO imbalance also contributes to vascular remodeling and structural changes linked to cardiovascular diseases such as atherosclerosis (Slowinski et al., 2007). Akt, a central molecule in the PI3K/Akt pathway, activates eNOS, leading to increased NO production in endothelial cells (Higashi et al., 2009). Fulton et al. demonstrated that Akt activates eNOS by phosphorylation, independent of calcium influx, thereby increasing NO production. The PI3K/Akt pathway can induce eNOS phosphorylation and activation without requiring increased intracellular calcium (Fulton et al., 1999).

#### 5.1.4 Endothelial dysfunction and vasoconstriction

BTKi selectively targets BTK, inhibiting the normal signaling through the BCR and PI3K/Akt pathways (Qin et al., 2021). Although existing research has revealed the role of BTKi in modulating the BCR and PI3K/Akt pathways, the complex interactions between these pathways remain not fully understood, particularly in terms of their specific mechanisms in vascular function and blood pressure regulation. A potential mechanism for BTKi-induced hypertension is that the inhibition of BTK leads to a decrease in p-PI3K and p-Akt levels, reducing eNOS activation and suppressing NO synthesis (Farah et al., 2018; Xin et al., 2018). The reduction in NO production activates a negative feedback mechanism that increases ET-1 expression, further promoting vasoconstriction and inflammatory responses (Slowinski et al., 2007). The imbalance between NO and ET-1 results in endothelial dysfunction, causing abnormal reductions in vascular tone and vasospasm in smooth muscle cells (Gallo and Savoia, 2024). Increased vasoconstriction and inflammation contribute to elevated blood pressure, eventually leading to the development and progression of hypertension (Citrin et al., 2024; Hirase and Node, 2012).

### 5.2 MAPK signaling pathway

#### 5.2.1 Role of MAPK in regulating vascular smooth muscle cell (VSMC) proliferation

The MAPK signaling pathway is a crucial signaling cascade in cells, involved in essential physiological processes such as cell proliferation, differentiation, migration, survival, and stress responses (Fang and Richardson, 2005). The primary role of the MAPK pathway is to regulate cellular activities via a multistep kinase cascade. The MAPK signaling pathway is generally categorized into three primary subtypes: ERK, JNK, and p38 MAPK (Dhillon et al., 2007). Among these, the ERK/MAPK pathway plays a crucial role in regulating cell proliferation. This signaling pathway is activated downstream of various growth factor receptors, including the epidermal growth factor receptor (Dhillon et al., 2007). A large body of literature has emphasized the significant role of the MAPK signaling pathway in regulating vascular smooth muscle cell (VSMC) proliferation. In response to mechanical forces, protein kinase C and MAPKs are activated, which leads to increased expression of c-Fos and c-Jun genes and enhanced DNA-binding activity of the transcription factor AP-1, thus regulating the growth and response of VSMCs (Li and Xu, 2000). Xi XP and colleagues demonstrated that the MAPK pathway is not only crucial for VSMC growth induced by basic fibroblast growth factor (bFGF), but also essential for VSMC migration induced by platelet-derived growth factor (PDGF) (Xi et al., 1999). P38 MAPK influences VSMC metabolic status by promoting mitochondrial fragmentation. While mitochondrial fragmentation may reduce ATP production, it stimulates VSMC proliferation. P38 MAPK significantly regulates VSMC proliferation and angiogenesis through PGC-1α-dependent mitochondrial dynamics (Sahún-Español et al., 2022). SO_2_ inhibits the activation of the ERK/MAPK pathway through upstream signaling by increasing cellular cAMP levels, activating PKA, and promoting phosphorylation of c-Raf at Ser259, which prevents c-Raf activation. This inhibition of c-Raf halts the progression of the cell cycle and suppresses VSMC proliferation (Liu et al., 2014). Ang II and PDGF rapidly elevate intracellular Ca^2+^ concentrations in VSMCs, activating PKC and MAPK. However, Ang II is more likely to induce cell hypertrophy, while PDGF is more likely to stimulate cell proliferation (Liao et al., 1996). MAPK regulates VSMC growth and proliferation via diverse mechanisms. VSMCs are integral to the vascular wall, maintaining vascular structure and function (Li and Fukagawa, 2010). Their hypertrophy, proliferation, and migration are critical events in the development of atherosclerosis, thereby linking MAPK signaling with vascular structure and function (Xu et al., 1997).

#### 5.2.2 BTKi-mediated MAPK activation and hypertension

Currently, there are no direct studies investigating how BTKi affects the MAPK signaling pathway. However, research has indicated that BTKi can inhibit the MAPK pathway, reduce CD93-mediated immune suppression, and enhance the antitumor effect of T cells (Yu YQ et al., 2024). Additionally, MAPK pathway activation plays a pivotal role in B cell proliferation and survival, the development of BTKi resistance, and antitumor activity, suggesting that BTKi may impact the MAPK signaling pathway (Wang et al., 2021; Wang et al., 2022). Within the BCR signaling network, key molecules in the MAPK pathway, including ERK, JNK, and p38/MAPK, can be directly phosphorylated by PKCβ, thus activating the MAPK signaling pathway (Jacob et al., 2002). The key substance that activates PKCβ, IP3, is produced by the hydrolysis of PIP2 by PLC-γ2, with PLC-γ2 activation closely linked to BTK (Pal et al., 2018; Engels et al., 2001). By inhibiting BTK, BTKi prevents PLC-γ2 activation, thereby hindering MAPK pathway activation. Since MAPK plays an essential role in VSMC proliferation, BTKi-induced disruption of this pathway impairs VSMC proliferation, causing vascular dysfunction, decreased vascular tone, and ultimately contributing to elevated blood pressure (Bennett et al., 2016; Lacolley et al., 2012).

### 5.3 NF-κB signaling pathway

#### 5.3.1 The link between the NF-κb signaling pathway and the immune system

The NF-κB signaling pathway operates through two main routes: the classical and non-classical pathways (Mulero et al., 2019). The classical pathway is activated by inflammatory cytokines such as TNF-α and IL-1β, which rapidly trigger NF-κB activation, primarily through the IKK complex (Vallabhapurapu et al., 2008). The IKK complex phosphorylates IκB (NF-κB inhibitor), causing its dissociation and the release of NF-κB dimers (such as p65/RelA and p50), which then promote the transcription of target genes (Taniguchi and Karin, 2018). The activation of the non-classical NF-κB pathway occurs more gradually, as it involves the *de novo* synthesis of NF-κB-inducing kinase (NIK, or MAP3K14) (Sun, 2017). This pathway is triggered by specific cytokines from the TNF family, including lymphotoxin (LT), receptor activator of NF-κB ligand (RANKL, also referred to as TNFSF11), CD40 ligand (CD40L), and B-cell activating factor (BAFF, or TNFSF13B) (Bonizzi and Karin, 2004). NF-κB regulates the expression of various genes that are integral to innate immune responses. These genes include those that encode for various cytokines (such as IL-1, IL-2, IL-6, IL-12, TNF-α, LTα, LTβ, and GM-CSF), adhesion molecules (like intercellular adhesion molecule, vascular cell adhesion molecule, and endothelial leukocyte adhesion molecule), chemokines (including IL-8), acute-phase proteins (e.g., SAA), and inducible enzymes (such as iNOS and COX-2) (Ghosh et al., 1998; Caamaño and Hunter, 2002; May and Ghosh, 1998). NF-κB plays a crucial role in modulating immune responses, particularly in activating immune cell functions such as differentiation, proliferation, and survival, and is essential for both innate and adaptive immunity (Mulero et al., 2019). In the adaptive immune response, T-cells and B-cells play crucial roles as key components of the system (Golubovskaya and Wu, 2016). Upon activation, both T- and B-cells undergo processes of proliferation and differentiation, evolving into effector cells that perform various immune functions. These include cytokine secretion and the cytotoxic T-lymphocyte (CTL) response in T-cells, as well as the production of antibodies by B-cells (O'Shea and Paul, 2010). Additionally, some of the activated lymphocytes differentiate into long-lived memory cells, which enable a swift and enhanced immune response upon subsequent encounters with the same pathogen (Krebs and Steinmetz, 2016). The non-classical NF-κB signaling pathway is critical in B cell development, function, and immune responses. It directly affects B cell survival, proliferation, and class-switch recombination. It ensures the proper functioning of B cells in antigen-specific immune responses by regulating germinal center (GC) reactions, cytokine production, and immune response progression (Gatto and Brink, 2010; De Silva et al., 2016). In peripheral tissues, the non-classical NF-κB pathway significantly affects T cell survival, differentiation, and the maintenance of memory T cells (Boehm and Swann, 2013). A deficiency in the non-classical NF-κB pathway disrupts T cell memory responses, especially during the generation and maintenance of memory T cells (Thapa and Farber, 2019). Mice deficient in NIK show significant impairment in memory T cell responses and fail to mount effective responses to viral reinfection (Rowe et al., 2013). NF-κB is essential in immune responses through both classical and non-classical pathways, working synergistically to ensure effective antigen recognition, immune response, and the establishment of long-term immune memory.

#### 5.3.2 BTKi-mediated immunosuppression leads to abnormal vascular function

BTK is involved in regulating several signaling pathways in immune cells, particularly in B cells and myeloid cells. Beyond its central role in BCR signaling, BTK is also closely linked to the signaling of various receptors, such as BAFFR, CD40, Fc receptors, and GPCRs (Neys et al., 2021). Research has demonstrated that BAFFR activates the typical NF-κB signaling pathway through crosstalk with the BCR signaling pathway, which involves SYK and BTK (Shinners et al., 2007). CD40, a co-stimulatory receptor, is integral to immune cell activation. BTK regulates CD40 signaling *via* the non-classical NF-κB pathway and other mechanisms, ultimately promoting B cell survival, differentiation, and proliferation (Brunner et al., 2002; Schwartz et al., 2014). BTK is also a crucial component of the FcR signaling pathway (Owens et al., 2022). RANKL (Receptor Activator of NF-κB Ligand) regulates osteoclast differentiation and development based on BTK-mediated FcR signaling (Watterson et al., 2019; L Deng et al., 2018). BTK regulates NF-κB signal transduction activity through interactions of a variety of receptor signal transduction pathways and plays a pivotal role in the survival, differentiation, proliferation, and response of the immune cell. As a coordinator, BTK ensures the immunity cell’s ability to respond to a variety of physiological and pathological conditions. Effective NF-κB signal transduction depends on the proper functioning of the pathway. The NF-κB signal transduction pathway plays a pivotal role in the development, differentiation, proliferation, and activation of the immune cell (Sun, 2017). By regulating the production of cytokines, the activation of immune cells, and recognition of antigens, the NF-κB signal pathway ensures the immune function of combating external pathogens and ensuring the establishment of immunotolerance in order to avert autoimmune disease (Deng et al., 2018). Inhibiting the activity of BTK by BTKi thus may cause the downregulation of NF-κB signal pathways that could potentially bring about immunosuppression (Song et al., 2024). Agents of immunosuppression have been the subject of extensive studies as a means of treatment after organ transplantation (Kadosawa and Watabe, 2015). Although such agents have great benefits of lowering organ rejection, they also have a detrimental impact on the vascular endothelial cells by impairing endothelial-dependent relaxation as well as smooth muscle cell responsiveness towards a variety of vasoconstrictors like Ang II (Petrakopoulou et al., 2006; Mathew et al., 2011). Studies indicate that cyclosporine A, a widely used immunosuppressant, can induce endothelial injury, accompanied by increased levels of ET-1 and PGI2 (Wilasrusmee et al., 2003). BTKi, by blocking the BTK signaling pathway, may suppress immune activity through NF-κB pathway inhibition, further impairing the vascular endothelium and potentially contributing to elevated blood pressure.

### 5.4 Notch signaling pathway

#### 5.4.1 Notch’s role in vascular development and hypertension

The Notch signaling pathway is a highly conserved molecular cascade that regulates various cellular processes, including growth, differentiation, and pattern formation (Aquila et al., 2019). In mammals, the pathway is defined by four transmembrane receptors (Notch 1-4), which interact with five typical transmembrane ligands (Jagged 1, 2; Delta-like ligands 1, 3, and 4) (Aquila et al., 2019). The Notch signaling pathway can be classified into classical and non-classical pathways. The classical pathway primarily relies on ligand binding to the NOTCH receptor, which triggers endocytosis and a series of cleavage events (S1, S2, S3 cleavages), ultimately regulating gene transcription *via* NICD (Guo et al., 2024). The non-classical pathway is more dynamic, capable of activating Notch signaling in a ligand-independent manner, bypassing the conventional endocytosis process. This pathway interacts more extensively with extracellular and intracellular signaling networks, expanding the biological functions of Notch signaling (Zhou et al., 2022). There is growing evidence that Notch receptors and downstream Notch effectors play essential roles in both embryonic and postnatal vascular development and the response of vascular smooth muscle cells to growth factor stimulation and vascular wall injury (Hasan et al., 2017). Notch signaling plays a critical role in angiogenesis by interacting with its ligands and engaging in crosstalk with other key pathways, such as the vascular endothelial growth factor (VEGF) signaling pathway. This interaction is essential for the regulation of blood vessel formation (Siebel and Lendahl, 2017). Delta-like ligand 4 (DLL4) is identified as a key Notch ligand that promotes angiogenesis (Akil et al., 2021). Notch signaling coordinates the tip and stalk cell phenotypes during angiogenesis in growing sprouts. DLL4 expression in tip cells activates Notch1 in stalk cells. The interaction between VEGF and Notch signaling is crucial for several processes, including the formation of sprouts, the survival of endothelial cells, and the establishment of cellular diversity within the vasculature (Blanco and Gerhardt, 2013). Mutations in Notch receptors and ligands in mice lead to abnormalities in multiple tissues, including the vascular system, as seen in Alagille syndrome (AGS) and autosomal dominant cerebral arteriopathy with subcortical infarcts and leukoencephalopathy (CADASIL), which are caused by mutations in the Notch ligand Jagged1 and the Notch3 receptor, respectively (Joutel et al., 1996; Li L. et al., 1997). These findings highlight the critical role of the Notch pathway in vascular development.

The Notch signaling pathway is widely implicated in pulmonary hypertension. Notch3 expression is associated with the progression of pulmonary hypertension. Studies conducted at various time points during hypoxia-induced (in mice) or methylene blue-induced (in rats) pulmonary hypertension models showed an increase in Notch3 expression in the lungs as a function of time and disease severity. The research found that, compared to animals in 21% oxygen, mice exposed to 6 weeks of hypoxia had three times higher levels of Notch3 expression at both the mRNA and protein (ICD) levels in their lungs, with pulmonary artery pressures consistent with late-stage pulmonary hypertension (Thistlethwaite et al., 2010). The use of the Notch signaling inhibitor DAPT significantly reduced the thickness of the pulmonary artery vessel wall and induced apoptosis in vascular smooth muscle cells, as observed in the study. This indicates that Notch signaling plays a significant role in vascular remodeling in pulmonary arterial hypertension (PAH) (Qiao et al., 2012). In patients with idiopathic pulmonary arterial hypertension (IPAH) and the hypoxia/SU5416 (SUHx) rat model, Notch1 expression was markedly increased, particularly in pulmonary arterial endothelial cells. Notch1 expression positively correlated with endothelial cell proliferation markers, such as PCNA. When Notch1 signaling was inhibited using γ-secretase inhibitors (DBZ), there was a significant reduction in the proliferation and migration of pulmonary artery endothelial cells (PAECs), along with suppression of VEGF-induced endothelial cell responses, suggesting that Notch1 is crucial for endothelial cell proliferation (Del Papa et al., 2019). The Notch signaling pathway has been identified as an important target in regulating pulmonary hypertension, particularly in the processes of vascular remodeling, endothelial cell proliferation, and apoptosis (Morris HE. et al., 2019). Therefore, targeting Notch signaling may offer a promising therapeutic approach for pulmonary hypertension, and further clinical studies and treatment trials will be essential to validate this hypothesis (Smith et al., 2015; Li et al., 2009).

#### 5.4.2 How BTKis interfere with Notch signaling in endothelial cells

Currently, no studies specifically examine how BTKi regulates the Notch signaling pathway. However, recent literature has reported that key molecules in the Notch signaling pathway experience reduced activity after BTKi treatment. For example, Wang W and colleagues indicated that the clinical efficacy of ibrutinib is associated with a downregulation of Notch1 activity, which becomes more pronounced over time (Wang et al., 2024). Other studies have shown an interaction between BTK and Notch1, with BTKi inhibiting Notch signaling. Moreover, Notch1, a new molecular partner in the BCR signaling pathway, offers potential for improving targeted therapies. *In vitro* studies have shown that ibrutinib significantly reduces Notch1/2 activation and induces the dephosphorylation of eIF4E (a Notch1 target gene) (Del Papa et al., 2019). In CLL patients, the Notch1 signaling pathway is gradually inhibited after ibrutinib treatment but is restored during disease relapse (Smith and Burger, 2021; Liu et al., 2022; Nakhoda et al., 2023). These reports suggest crosstalk between the Notch and BTK signaling pathways, meaning that inhibiting BTK signaling with BTKi leads to a decrease in Notch pathway activity. However, these pieces of evidence are derived from a limited number of clinical studies, and relevant research remains scarce, resulting in considerable uncertainty. Moreover, no molecular experiments have yet demonstrated how BTK signaling regulates Notch signaling.

### 5.5 RhoA/ROCK signaling pathway

#### 5.5.1 RhoA/ROCK pathway in vascular tone regulation

RhoA, a member of the Rho family, is regulated by guanosine triphosphate (GTP) binding. It cycles between its active form, GTP-bound, and its inactive form, GDP-bound (Li et al., 2022). ROCK (Rho-associated coiled-coil containing kinase) is a downstream effector of RhoA, acting as a Rho-GTPase-activated serine/threonine kinase. It regulates LIM domain kinases (Lin11, Is-1, and Mec-3), myosin light chain (MLC), and MLC phosphatase, playing an essential role in promoting actin cytoskeleton contraction (Somlyo et al., 2000; Kaneko et al., 2002). The signaling pathway mediated by RhoA/ROCK, along with its interactions with Ang II, oxidative stress, and NO, is integral to the pathogenesis of cardiovascular diseases (Seccia et al., 2020). Activation of the RhoA/ROCK pathway increases calcium sensitivity and vascular tone, leading to alterations in cardiovascular and renal structures (Calò and Pessina, 2007). ROCK2 enhances myosin phosphorylation and vasoconstriction through direct binding with BMAL1 (aryl hydrocarbon receptor nuclear translocator-like) (Xie et al., 2015). By upregulating RhoA protein and preventing its degradation, BMAL1 further promotes ROCK-mediated stress fiber reorganization and the formation of the actin cytoskeleton, intensifying vasoconstriction (Ma et al., 2018). ROCK also phosphorylates and inhibits phosphatase and tensin homolog (PTEN), which blocks the pro-survival PI3K pathway (Street and Bryan, 2011). Additionally, because the PI3K/Akt pathway promotes eNOS expression, ROCK-mediated PTEN activation reduces endothelial nitric oxide (NO) production and cell survival (Street and Bryan, 2011). In addition to lowering eNOS expression, the RhoA/ROCK signaling pathway also diminishes eNOS activity by inhibiting the phosphorylation of Ser1177 on eNOS. This results in a rapid decline in eNOS function (Ming et al., 2002). Overall, the RhoA/ROCK pathway not only contributes to endothelial dysfunction but also plays a role in endothelial cell activation. For instance, it enhances the expression of ICAM-1 and VCAM-1 on the endothelial surface via the NF-κB signaling pathway, thereby promoting leukocyte infiltration and vascular inflammation (Ming et al., 2002). In addition to endothelial cells, the RhoA/ROCK signaling pathway mediates vascular smooth muscle cell dysfunction (VSMCs). For instance, a study by Guilluy et al. demonstrated that Ang II promotes vascular smooth muscle cell (VSMC) contraction, which contributes to increased blood pressure. This process is mediated by the activation of RhoA guanine nucleotide exchange factor ARHGEF1 by Ang II, leading to the subsequent activation of RhoA (Guilluy et al., 2010). Moreover, the association between the RhoA/ROCK pathway and elevated blood pressure has been supported by indirect evidence from pharmacological interventions. In animal models of hypertension induced by soluble factors such as Ang II or L-NAME (L-Nitro-Arginine Methyl Ester), ROCK inhibitors successfully lowered blood pressure. It alleviated hypertension-associated vascular inflammation and remodeling (Shiga et al., 2005; Löhn et al., 2009).

The RhoA/ROCK signaling pathway is involved in the regulation of actin cytoskeleton contraction, cell migration, and vascular tone. Activation of this pathway plays a critical role in the development of cardiovascular diseases through its interactions with Ang II, oxidative stress, and NO. Specifically, RhoA/ROCK signaling contributes to endothelial dysfunction and vascular remodeling by increasing calcium sensitivity, enhancing vasoconstriction, promoting stress fiber reorganization, and inhibiting endothelial nitric oxide synthesis. Clinical studies and drug interventions in animal models suggest inhibiting the RhoA/ROCK signaling pathway could mitigate vascular inflammation, prevent remodeling, and reduce high blood pressure. This makes it a promising target for treating hypertension and related cardiovascular diseases.

#### 5.5.2 Potential BTKi-mediated dysregulation contributing to hypertension

Although there is no direct evidence linking BTK to regulating the RhoA/ROCK pathway, a potential interaction exists between the two. They may cooperatively regulate cellular functions through mutual modulation in specific contexts, especially in immune cells and vascular functions. Recent studies have demonstrated that RhoA is a crucial participant in immune cell responses, influencing the migration and activation of both innate and adaptive immune cells (Kilian et al., 2021). The RhoA/ROCK pathway regulates cytoskeletal rearrangement in B cell compartments (Saci and Carpenter, 2005). *In vitro* studies of BCR dynamics suggest that active RhoA limits BCR mobility by affecting the actin-severing protein cofilin, thereby interfering with the capacity of TLR ligands to enhance BCR signaling (Freeman et al., 2015). BTK is a critical kinase in the BCR signaling pathway, playing a key role in normal B cell development and proliferation (Mouhssine et al., 2024). These pathways work together to maintain the typical structure and function of B cells, and they can interact with shared signaling pathways, such as the NF-κB pathway (Smith and Burger, 2021; Xie et al., 2013).

**FIGURE 2:**
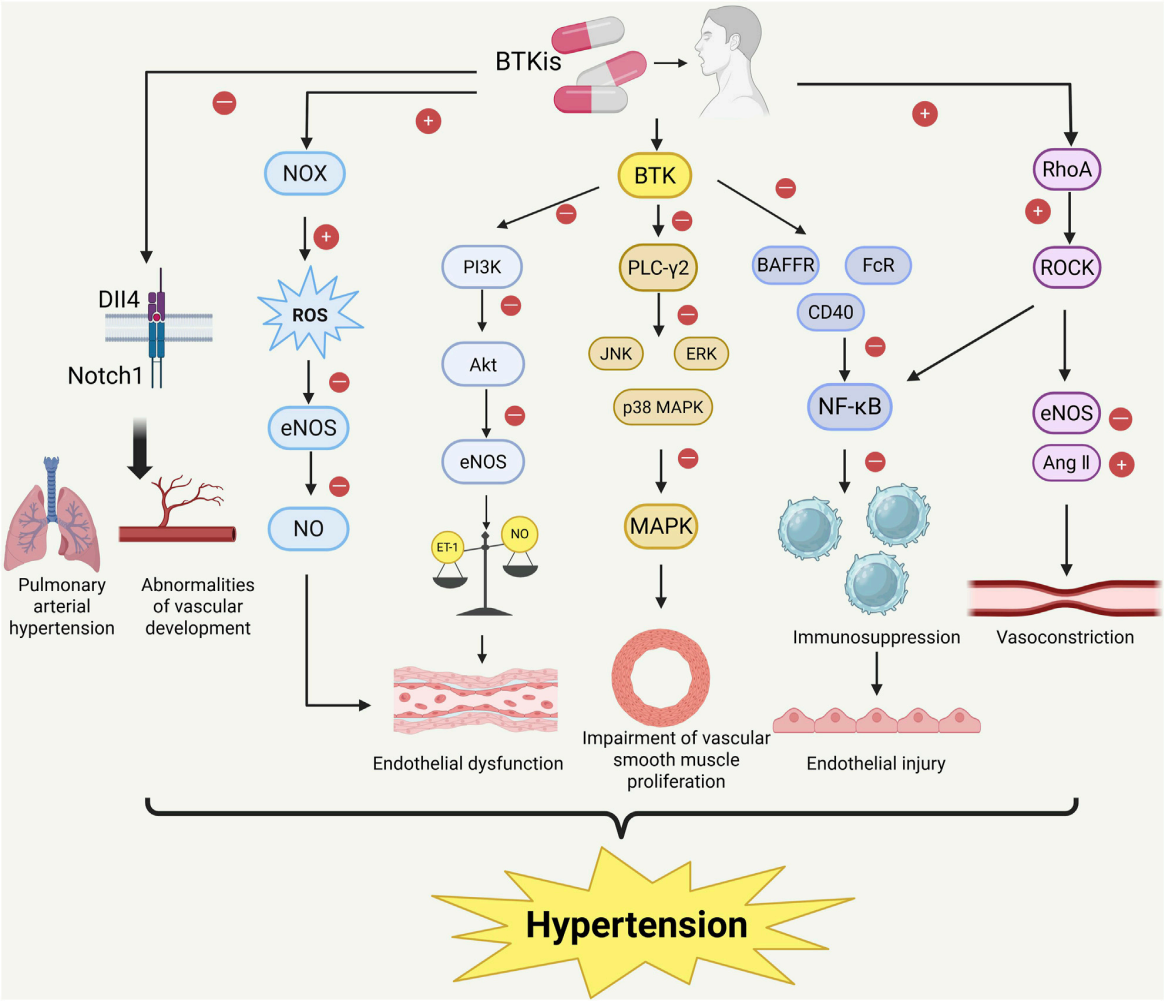
Proposed mechanisms of BTKi-induced hypertension. Bruton tyrosine kinase inhibitors (BTKis) may promote the development of hypertension through a variety of interconnected signaling pathways. BTKi inhibition promotes the production of reactive oxygen species (ROS) through NADPH oxidase (NOX) activation, resulting in decreased endothelial nitric oxide synthase (eNOS) activity and decreased nitric oxide (NO) utilization, which triggers endothelial dysfunction. In addition, BTKI-mediated inhibition of PI3K/Akt pathway reduces eNOS activity and disrupts the balance between endothelin-1 (ET-1) and NO, further aggravating endothelial dysfunction. Inhibition of downstream MAPK signaling and NF-κB activation leads to impaired vascular smooth muscle proliferation and immunosuppression. BTKi treatment may also affect vascular homeostasis by affecting the Notch1/Dll4 signaling axis, leading to vascular dysplasia and pulmonary hypertension. Finally, activation of the RhoA/ROCK pathway leads to enhanced smooth muscle contraction and vascular remodeling by reducing eNOS activity and enhancing angiotensin II (AngII) signaling. These mechanisms work together to eventually lead to endothelial dysfunction, vascular remodeling, and the development of hypertension. Image created with BioRender.com (accessed on 9 May 2025).

BTK may affect RhoA activity by activating the NF-κB pathway, which then promotes ROCK activation, leading to increased vascular smooth muscle contraction and vascular remodeling, thereby raising vascular tension. Additionally, BTKi, by inhibiting BTK activity, may cause an imbalance in the regulation of both the immune and vascular systems, contributing to hypertension. Specifically, BTKi treatment may lead to abnormal activation of the RhoA/ROCK signaling pathway, exacerbating smooth muscle cell contraction and consequently elevating blood pressure. However, this is merely a hypothesis based on some indirect evidence, and whether the RhoA/ROCK signaling pathway is truly involved in BTKi-mediated hypertension remains to be confirmed by further studies in the future. (Figure 2).

## 6 Clinical implications and management strategies

### 6.1 Monitoring blood pressure in patients receiving BTKis

Monitoring blood pressure is an essential aspect of managing BTKi treatment, especially for patients with a history of cardiovascular disease or hypertension. Regular monitoring enables clinicians to identify potential cardiovascular issues early and make timely adjustments to the treatment regimen, ensuring safety and therapeutic efficacy. In clinical practice, blood pressure monitoring is generally classified into office blood pressure measurements and out-of-office blood pressure monitoring.

Blood pressure measurements typically used for screening hypertension and adjusting antihypertensive treatment are often taken in a clinical setting and are commonly referred to as office blood pressure measurements. Blood pressure can be measured manually with an aneroid sphygmomanometer and a stethoscope to listen for Korotkoff sounds or with an automatic blood pressure monitor. Typically, medical assistants or nurses conduct these measurements (Muntner et al., 2019a). The procedure requires the patient to rest for 3–5 min before the measurement, which should be performed in a quiet environment. The patient’s legs should be flat on the floor, the back supported (as examination tables are generally not ideal), and the arm positioned at heart level. The cuff must be appropriately sized and placed on the bare arm, ensuring the bladder is empty. Additionally, the patient should refrain from consuming caffeine or smoking for at least 30 min before the measurement (Kallioinen et al., 2017). At least two additional measurements should be taken during the visit for individuals with elevated office blood pressure readings, as multiple readings often improve accuracy. Treatment decisions are based on the average of three office measurements (Muntner et al., 2019b; Whelton et al., 2017). Given the elevated risk of vascular toxicity and thromboembolic events associated with various chemotherapy and cancer therapies, it is recommended that patients have their inter-arm blood pressure difference checked at least once during treatment and afterward. If a consistent difference of 10 mmHg or more is found in either systolic or diastolic pressure between the arms, the arm with the higher pressure should be used for subsequent measurements (Muntner et al., 2019b).

Out-of-office blood pressure measurements address many of the limitations associated with clinical blood pressure measurements (Cohen and Cohen, 2016). Measuring blood pressure outside of the clinical setting is particularly useful for identifying conditions such as white coat hypertension (where office blood pressure is elevated but out-of-office readings are normal) and masked hypertension (where office blood pressure appears normal but out-of-office readings are elevated) (Cohen et al., 2019b). Studies indicate that both white coat and masked hypertension are more frequently observed in patients undergoing cancer treatment than in the general population (Cohen et al., 2019b; Bamias et al., 2011). The increased prevalence of white coat hypertension is thought to be related to the high anxiety and fear of prognosis associated with cancer diagnosis. The higher prevalence of masked hypertension may be partly due to the delayed onset of adverse effects from cancer treatments. Measurements are typically taken every 30–60 min, day and night. Ambulatory blood pressure monitoring is considered the gold standard in blood pressure measurement, as it correlates more strongly with cardiovascular outcomes than traditional clinical blood pressure readings (Cohen and Cohen, 2016). Home blood pressure monitoring usually requires the patient to take two measurements twice daily for at least 3 days (ideally 5–7 days) using a semi-automatic blood pressure monitor. While home monitoring can sometimes be less accurate than clinical measurements, these issues can be addressed with proper patient education on measurement techniques (McManus et al., 2014). Recent guidelines advise that all patients with an office blood pressure reading of 120/70 mmHg undergo 24-h ambulatory blood pressure monitoring. This approach helps more accurately assess blood pressure patterns outside the clinic (Whelton et al., 2017). Given the pharmacokinetics of most antihypertensive medications, it is typically recommended that patients begin home monitoring at least 3 days (ideally 5–7 days) after changing their treatment regimen. For patients with severe or symptomatic hypertension, earlier monitoring should be considered (Cohen et al., 2019a).

### 6.2 Potential therapeutic interventions

There are currently no specific guidelines for managing hypertension during BTKi therapy. According to the 2022 ESC Cardio-Oncology Guidelines, ACE inhibitors (ACEI) or angiotensin II receptor blockers (ARB) are recommended as first-line treatments to reduce the risk of chemotherapy-related cardiovascular diseases (CTRCD). For cancer patients with a systolic blood pressure (SBP) ≥160 mmHg and diastolic blood pressure (DBP) ≥100 mmHg, a combination of ACEI or ARB with a dihydropyridine calcium channel blocker (CCB) is recommended, as this combination provides faster blood pressure control compared to monotherapy with ACEI/ARB. If severe hypertension is diagnosed (SBP ≥180 mmHg or DBP ≥110 mmHg), a multidisciplinary team (MDT) should assess the competing risks of cancer and cardiovascular diseases (CVD). Any cancer treatment associated with hypertension should be postponed or temporarily stopped until blood pressure is controlled to below 160/100 mmHg. Once blood pressure is under control, cancer treatment can be restarted, and consideration should be given to dose reduction. For patients with drug-resistant hypertension related to cancer treatment, options such as spironolactone, oral or transdermal nitrates, and hydralazine should be considered. Beta-blockers like carvedilol or nebivolol may be appropriate for those exhibiting signs of high sympathetic nervous activity, stress, or pain. Diuretics, especially spironolactone, can be beneficial for cancer patients with hypertension and evidence of fluid retention. However, it is crucial to closely monitor blood pressure, electrolytes, and renal function when using these medications due to the potential for increased fluid retention. The decision to initiate antihypertensive treatment and select target therapies depends on the cancer condition and prognosis (Lyon TL and Couch, 2022; B Williams et al., 2018). Additionally, regular exercise, reducing alcohol intake, limiting sodium consumption, improving dietary habits, and stress reduction techniques along with improved sleep hygiene can enhance blood pressure control and potentially help prevent the development of hypertension (B Williams et al., 2018; T Unger et al., 2020; Whelton RMC et al., 2017; Valenzuela et al., 2021).

**FIGURE 3:**
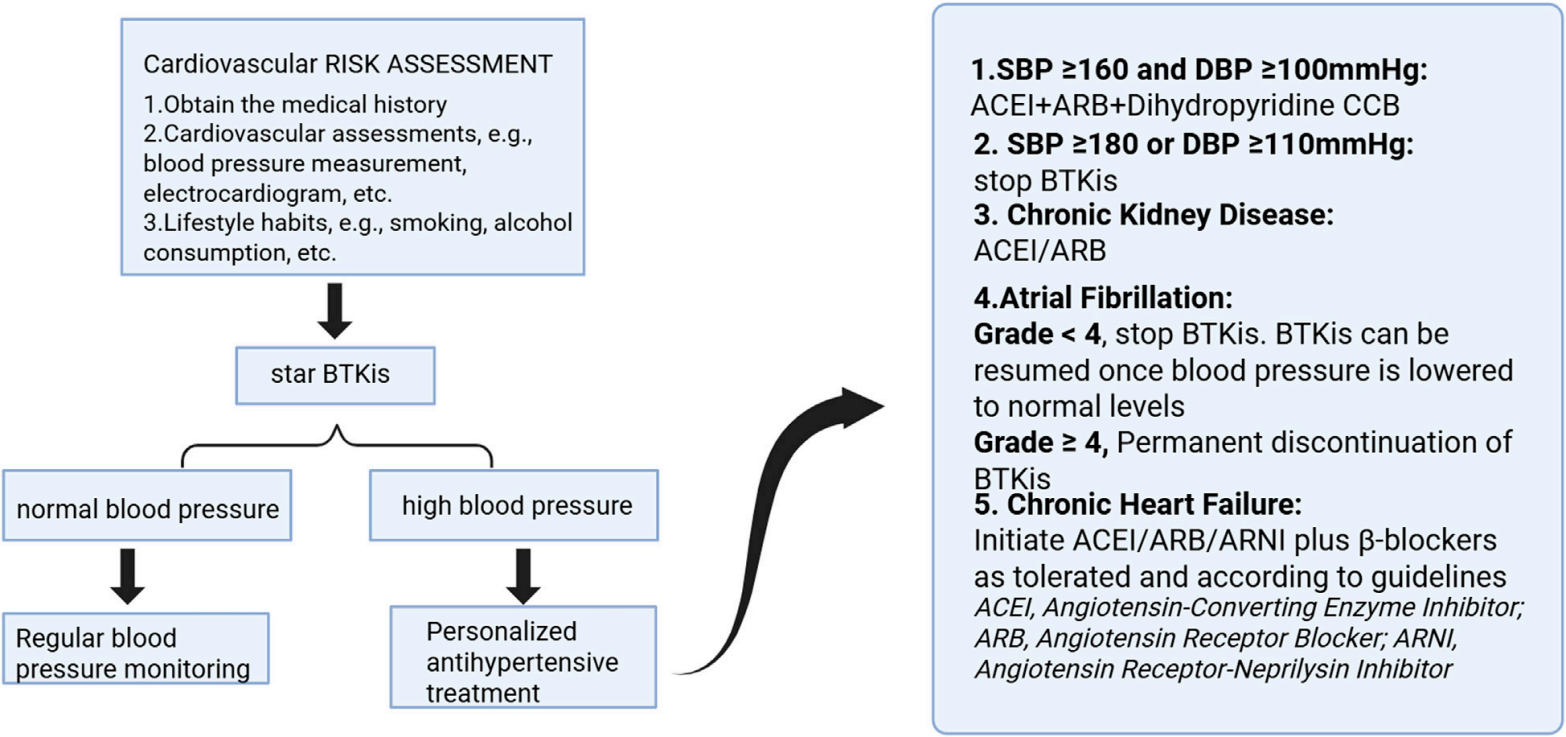
Management strategies for complications during BTKis treatment. Image created with BioRender.com (accessed on 9 May 2025).

Several clinical trials have explored the combination of BTKi with antihypertensive medications. A study by Samples et al. found that for hypertensive patients (whether they had pre-existing hypertension or were newly diagnosed with hypertension after treatment), no single antihypertensive drug significantly lowered mean arterial pressure (MAP). In particular, calcium channel blockers (CCBs) did not show significant effectiveness in reducing MAP and might even slightly raise MAP. The combination of ACE inhibitors (ACEI) or angiotensin II receptor blockers (ARBs) with hydrochlorothiazide (HCTZ) significantly reduced MAP. These combinations also effectively maintained normal blood pressure (with MAP below 120/80 mmHg). Multidrug combination therapy played a critical role in managing BTKi-induced hypertension. For patients with a history of hypertension, the combination of beta-blockers (BBs) and HCTZ proved more effective. In contrast, for those with newly diagnosed hypertension, the combination of ACEI/ARBs and HCTZ was more suitable (Samples et al., 2024). Mazyar Shadman and colleagues discovered that in patients with pre-existing hypertension (pre-HTN), the combination of HCTZ and beta-blockers (BB) significantly reduced mean arterial pressure (MAP). In patients with *de novo* hypertension (*de novo* HTN), the combination of HCTZ with ACE inhibitors (ACEI) or angiotensin II receptor blockers (ARB) also significantly lowered MAP. Furthermore, for both groups of patients, the most substantial reduction in MAP occurred when three or more antihypertensive medications were used. Quadruple therapy showed a particularly significant effect (M Shadman et al., 2022). Chen ST and colleagues found that although some patients started antihypertensive medications during treatment, no single medication was effective in preventing the worsening of hypertension. Similar results were reported by Dickerson T and colleagues (Dickerson et al., 2019). These findings suggest that combination therapy could become an emerging trend in the treatment of BTKi-induced hypertension (Figure 3).

### 6.3 Need for personalized approaches in hematologic malignancies

There may be considerable differences in the presentation, progression, drug resistance, and treatment response of diseases among individual patients. As a result, developing a personalized treatment plan based on the specific characteristics of each patient is crucial to improving efficacy, reducing side effects, and enhancing prognosis. Considering the varying risks of cardiovascular adverse events in patients receiving BTKi therapy, hematologists should evaluate each patient’s cardiovascular risk before and during treatment and establish individualized preventive strategies. It is critical to have a clear understanding of the patient’s medical history before initiating BTKi treatment, including a history of arrhythmias, hypertension, and a family history of sudden death (Cohen et al., 2019a). Additional cardiovascular evaluations, such as blood pressure measurements, electrocardiograms (ECGs), echocardiography, and left ventricular ejection fraction assessment, should be performed. The pre-treatment evaluation will significantly impact the selection of antihypertensive drugs. Patients who have recently experienced a myocardial infarction or been diagnosed with heart failure are often prescribed ACE inhibitors (ACEI)/angiotensin II receptor blockers (ARB) and beta-blockers (BB), as these drugs are well-known for reducing morbidity and mortality (Whelton et al., 2017; Collet et al., 2021). Some clinicians may prescribe ACEI/ARB for all chronic kidney disease patients, as studies suggest that patients with severe proteinuria have a lower risk of progressing to end-stage renal disease, particularly among diabetic patients and those with excessive proteinuria (Sarafidis et al., 2007). For diabetic patients and those with significant proteinuria, ACEI/ARB are considered appropriate first-line options, while thiazide diuretics can adversely affect glucose metabolism (Hall et al., 2020). Patients with a tendency for orthostatic hypotension generally avoid diuretics. For patients with atrial fibrillation, BBs or CCBs are commonly used as part of a dual strategy for rate control (Samples et al., 2024).

Treatment and management measures for cardiovascular complications during BTKi therapy differ based on the severity of the condition. If atrial fibrillation is diagnosed in a patient receiving BTKi treatment, urgent cardiology consultation and management are recommended. For atrial fibrillation of grade <4, BTKi should be temporarily discontinued, while for grade 4 atrial fibrillation, BTKi should be permanently discontinued. Suppose ibrutinib is discontinued due to grade 3 or lower atrial fibrillation. In that case, therapy may be resumed once the atrial fibrillation resolves, with the dose reduced to 280 mg per day, or an alternative BTKi may be considered. The management of atrial fibrillation involves rhythm control, anticoagulation therapy (to prevent stroke), and the management of cardiovascular comorbidities (Cohen et al., 2019a). In the case of grade 2 heart failure (HF) during treatment, immediate treatment based on the CTCAE criteria is required. Once the adverse event improves to grade 1 or baseline, ibrutinib should be interrupted, and dosage adjustments should be made according to guidelines before restarting therapy. If grade 3 or 4 heart failure (severe or life-threatening) occurs, ibrutinib should be discontinued (Aghel et al., 2023). The management of hypertension has been detailed earlier.

Patients who present with sudden palpitations, shortness of breath, chest pain, edema, unexplained syncope, or elevated blood pressure should be urgently referred to a cardiologist for evaluation (Quartermaine et al., 2023).

By conducting a comprehensive cardiovascular risk assessment and implementing tailored treatment and preventive measures, cardiovascular adverse events linked to BTKi treatment can be significantly reduced, ensuring improved safety and efficacy of the therapy.

## 7 Future perspectives and research directions

### 7.1 Gaps in understanding BTKi-induced hypertension

The molecular mechanisms responsible for BTKi-induced hypertension remain unclear. Although some clinical observations suggest a connection between BTKi use and the onset of hypertension, the specific pathophysiological processes have not been fully understood (Jiang et al., 2024; Brem and O'Brien, 2022). Hypertension may involve several biological processes, including vasoconstriction, endothelial dysfunction, and kidney damage, but these mechanisms’ exact manifestation and role during BTKi treatment remain uncertain (Anyfanti et al., 2020). The understanding of how BTKi influences these physiological processes is still in its early stages, and the effects on organ systems such as the vasculature, heart, and kidneys have yet to be systematically explored.

Individual differences in genomics, metabolism, immune responses, and other factors may contribute to significant variations in blood pressure responses during BTKi treatment (Chen et al., 2022; Mouhssine et al., 2024). Most current studies overlook personalized factors such as genetic polymorphisms, variations in drug-metabolizing enzymes, and underlying cardiovascular diseases. Different individuals have varying metabolic processes and tolerances to BTKi, and these factors may play an essential role in the onset and progression of hypertension. Therefore, the limited focus on pharmacogenomics, immunogenetics, and cardiovascular individual differences has restricted a deeper understanding of the mechanisms behind BTKi-induced hypertension.

Although some clinical research has suggested a preliminary connection between BTKi treatment and hypertension, our understanding of its long-term effects remains inadequate (Tam and Thompson, 2024; Bennett et al., 2023; Sestier et al., 2021). Hypertension is often gradual, but most existing studies focus on short-term observations, with insufficient long-term follow-up data (Lipsky and Lamanna, 2020). The persistence of hypertension and its long-term consequences on the cardiovascular system (such as stroke, heart disease, kidney damage, etc.) have not been fully explored. Consequently, the lack of large-scale, multicenter, long-term data hinders a comprehensive evaluation of BTKi-induced hypertension.

### 7.2 Potential targets for mitigating cardiovascular risks in BTKi therapy

Oxidative stress plays a central role in cardiovascular diseases and may also be a contributing factor to the cardiovascular issues potentially induced by BTKi treatment. BTKi therapy might aggravate cardiovascular damage by enhancing oxidative stress. Antioxidants, such as N-acetylcysteine, vitamin C, and vitamin E, may alleviate oxidative stress, improve vascular function, and reduce the occurrence of hypertension (Gulcin, 2020; Baradaran et al., 2014; Amponsah-Offeh et al., 2023). Therefore, modulating oxidative stress levels could serve as an effective strategy to reduce cardiovascular risks during BTKi treatment.

Endothelial cells are critical in the regulation of blood vessel dilation and constriction (D Konukoglu, 2017). BTKi therapy may impair endothelial cell function, leading to vasoconstriction and elevated blood pressure. Research has demonstrated a strong correlation between endothelial dysfunction and the development of hypertension. Therefore, improving endothelial function, particularly by promoting NO synthesis or protecting endothelial cells from damage, may be a viable strategy for reducing cardiovascular risk (Wu et al., 2021; KK Griendling et al., 2021). Targeting endothelial-related signaling pathways, such as the NO/cGMP pathway, could help prevent BTKi-induced hypertension (Ataei Ataabadi et al., 2020).

Cardiovascular protective molecules such as brain natriuretic peptide (BNP) and cystatin C have been demonstrated to play a critical role in the progression of cardiovascular diseases (Z Cao and Zhu, 2019; L Qin and Li, 2020). Enhancing the expression or function of these molecules may help reduce cardiovascular adverse events induced by BTKi therapy. Developing therapeutic agents targeting these molecules could provide novel approaches for lowering cardiovascular risks during BTKi treatment.

Beta-receptors and calcium channels regulate cardiac contractility and smooth muscle contraction in blood vessels (L Qin and Li, 2020; O'Donnell, 2020; Zhu et al., 2021). Beta-blockers (such as metoprolol and amiodarone) and calcium channel blockers (such as diltiazem) are frequently used to treat arrhythmias, including atrial fibrillation (Denham et al., 2018; P Kirchhof and Goette, 2020). Research indicates that BTKi may increase the risk of atrial fibrillation by affecting cardiac structure and electrophysiology, particularly through atrial remodeling and fibrosis (JA Gambril et al., 2024). Therefore, targeting beta-receptors or calcium channels could effectively reduce the incidence of atrial fibrillation and lower cardiovascular risks.

### 7.3 Need for clinical trials and mechanistic studies

There is a notable gap in our understanding of the mechanisms that lead to hypertension in patients undergoing BTKi therapy. A significant portion of the research on BTKi is aimed at evaluating its anticancer efficacy and cancer prognosis in oncology clinical trials, where the hypertension data collected is limited, making in-depth analysis difficult (Butel-Simoes et al., 2023). Few clinical trials address pharmacological treatments for complications during BTKi therapy, which limits the ability of clinicians to manage these patients effectively. This underscores the urgent need for larger, higher-quality trials focused on hypertension, particularly antihypertensive treatment trials for cancer patients and survivors. Such trials could provide clinical guidelines and recommendations for screening and managing this unique patient population.

Research on the specific mechanisms by which BTKi therapy induces hypertension is currently insufficient. Our understanding of the molecular, cellular, and genomic-level pathophysiological processes involved in this side effect is still minimal. There is a pressing need for more high-quality preclinical models and research data to gain a deeper understanding of these mechanisms. These studies will show how BTKi affects blood pressure and offer theoretical guidance for more effective hypertension management in clinical practice.

Understanding the mechanisms through which BTKi therapy induces hypertension is of great importance. Hypertension is a common cardiovascular side effect in clinical practice, and if left unmanaged, it can lead to severe cardiovascular events, impacting the overall survival quality and prognosis of patients (Yin et al., 2024; Vallabhaneni et al., 2024). Gaining insight into these mechanisms will help identify patients more likely to develop hypertension, providing a foundation for personalized treatment and enabling the development of more effective preventive and intervention strategies. Additionally, detailed mechanistic research could offer valuable guidance for the future development of BTKi-like drugs, aiding in reducing cardiovascular side effects and improving the safety and efficacy of these medications. Therefore, enhancing research in this area is critical for managing hypertension in BTKi therapy, advancing cancer treatment, and improving patients’ quality of life.

## 8 Summary and conclusion

### 8.1 Summary of key findings

Bruton’s tyrosine kinase inhibitors (BTKis) have become pivotal in the treatment of hematologic malignancies, particularly B-cell lymphoma (BCL), chronic lymphocytic leukemia (CLL), small lymphocytic lymphoma (SLL), mantle cell lymphoma (MCL), and Waldenström macroglobulinemia (WM) (Jain et al., 2024; Shah et al., 2025; Wang et al., 2023; Treon et al., 2024). However, the clinical use of these inhibitors is frequently complicated by adverse reactions, with hypertension being one of the most notable side effects. The pathophysiological mechanisms behind BTKi-induced hypertension are not yet fully understood and require further investigation to optimize clinical treatment. This review explores the mechanisms of BTKi-induced hypertension, focusing on the key molecular pathways involved in this adverse effect.

Numerous clinical trials have indicated that patients receiving BTKi therapy, especially those treated with ibrutinib, have a high incidence of hypertension (Caldeira et al., 2019). Studies show that 25%–68% of patients develop new or worsened hypertension during treatment, and long-term follow-up research has confirmed that BTKi-induced hypertension increases the risk of major adverse cardiovascular events (MACE) associated with it (Dickerson et al., 2019; Samples et al., 2024). Taking prompt and appropriate actions when abnormal blood pressure is detected can significantly improve outcomes.

Hypertension during BTKi therapy may arise from multiple factors, including direct and indirect mechanisms. BTKi-induced oxidative stress plays a significant role in the onset and progression of hypertension by influencing various mechanisms that affect vascular tone and endothelial function (Xu et al., 2021). BTKi treatment has increased ROS levels, leading to the uncoupling of eNOS, significantly decreasing NO bioavailability (Honda et al., 2012). Decreasing the levels of NO inhibits vasodilation, augments vasoconstriction, and elevates blood pressure (Busse et al., 2002). The process involves NADPH oxidase, a mediator primarily regulated by BTK. Treatment of BTKi augments the activity of NADPH oxidase within immune cells, exacerbating oxidative stress throughout the body and resulting in endothelial malfunction (Honda et al., 2012). It not only impinges on the function of the immune cells but also extensively influences the health of the vessels and plays a role in the development of hypertension (Gallo and Savoia, 2024).

Along with oxidative stress, inhibition of BTK by BTKi will impair key pathways required for endothelial function, the most significant of which includes the PI3K/Akt pathway. The PI3K/Akt pathway plays a pivotal role in the activation of the production of NO by eNOS (Carrillo-Sepúlveda et al., 2010). Through the inhibition of the PI3K/Akt pathway, the activity of eNOS decreases by the BTKi, creating an imbalance between ET-1 and the production of NO, leading to endothelial dysfunction and the promotion of vasoconstriction (Byrd et al., 2021; Slowinski et al., 2007). The imbalance of vascular tone is a key contributor to the development of hypertension among subjects receiving treatment with BTKi.

The MAPK signaling pathway, a key pathway of cell proliferation, survival, and responses to stresses, was also modulated by the inhibition of the BTK (Yu YQ et al., 2024). The MAPK pathway, with the central players ERK, JNK, and p38 MAPK, regulates smooth muscle cell growth and migration. (Doronzo et al., 2011). Derangement of the MAPK signaling cascade impairs the function of vascular smooth muscle and contributes to endothelial malfunction as well as the development of hypertension (Liu et al., 2014).

Additionally, NF-κB signal transduction, a key role of which occurs under conditions of immune responses, contributes to the induction of hypertension by BTKi (A Alu et al., 2022). NF-κB activation supports the development and proliferation of immune cells and plays a pivotal role in maintaining immunofunction (L Deng et al., 2018). Under conditions of BTKi treatment, the blockade of BTK results in the modulation of the NF-κB signal transduction cascade, potentially leading to the induction of immunosuppression, deterioration of vascular function, and exacerbation of hypertension and enhancement of cardiovascular risk (Ahn and Brown, 2021).

The PI3K/Akt, MAPK, and NF-κB pathways are downstream of the BCR signal transduction pathway and are crucial for the development and function of B cells (JA Burger and Wiestner, 2018; Burger, 2019). The key kinase of the BCR signal transduction pathway, BTK, is reversibly or irreversibly occupied by the binding of BTKi at the Cys481 residue and induces the alteration of the downstream PI3K/Akt, MAPK, and NF-κB pathways, potentially leading to the development of hypertension (Gallo and Savoia, 2024). However, it is still unclear how these three pathways are suppressed in clinical scenarios, which of these pathways, or which combination, plays a dominant role in the occurrence of hypertension, and whether there are interactions between them. These uncertainties require further research to uncover the underlying mechanisms.

The Notch signaling pathway is critical in determining cell fate, tissue development, and differentiation. Disruption of Notch signaling can disturb the balance of endothelial cell proliferation, differentiation, and survival, leading to vascular instability and remodeling (Dabral et al., 2016). In BTKi treatment, inhibition of BTK may downregulate Notch, disrupting vascular homeostasis, increasing vascular resistance, and accelerating the development of hypertension in patients undergoing BTKi therapy (Del Papa et al., 2019). Furthermore, the RhoA/ROCK signaling pathway, which regulates smooth muscle contraction and cell migration, may also play a role in BTKi-induced hypertension. Activation of this pathway enhances smooth muscle contraction and promotes vascular remodeling, commonly seen in hypertensive patients (Löhn et al., 2009). However, there is currently limited direct evidence linking these two pathways to BTK, and the conclusions are largely uncertain. Therefore, more studies are recommended to explore the interactions between these pathways, which could provide additional targets for treating BTKi-induced hypertension.

This paper discusses treatment strategies to reduce BTKi-induced hypertension, with a particular emphasis on the role of combination therapy. While monotherapy has shown limited effectiveness in managing BTKi-induced hypertension, combination therapies—such as ACEI/ARB with HCTZ—have shown improved blood pressure control (M Shadman et al., 2022). Nevertheless, more extensive clinical trials are required to validate these approaches and refine treatment strategies.

In summary, the pathophysiology of BTKi-induced hypertension is multifactorial, involving intricate interactions between oxidative stress, endothelial dysfunction, and vascular signaling disruptions. Future research should prioritize further elucidating the molecular mechanisms behind BTKi-induced hypertension and investigating targeted treatments for its prevention and management. Personalized treatment approaches based on individual risk profiles are critical for minimizing cardiovascular risks and optimizing the efficacy and safety of BTKi therapy in treating hematologic malignancies.

### 8.2 Clinical relevance and importance of understanding hypertension in BTKi therapy

In section “3.2 Clinical Observations and Prevalence in Patients Receiving BTKi Treatment,” we have discussed that, according to numerous clinical studies, hypertension occurs at a specific incidence rate in patients treated with BTKi, regardless of the type of BTKi used. Hypertension is a significant risk factor for cardiovascular diseases and is closely related to the occurrence of cardiovascular complications such as stroke, myocardial infarction, and heart failure (Chen et al., 2022). Hypertension induced by BTKi therapy may increase the cardiovascular burden, particularly in patients with pre-existing cardiovascular conditions, who may face more severe cardiovascular events during BTKi treatment (Gordon et al., 2023).

Hypertension not only affects a patient’s overall health but also has the potential to impact the continuity and efficacy of BTKi treatment (I Goh and Chew, 2018). In some instances, severe hypertension may result in the interruption or adjustment of BTKi doses, which could compromise treatment outcomes (Sapkota et al., 2021). Thus, the timely identification and management of BTKi-induced hypertension are critical to ensuring both the safety and efficacy of therapy (L Ar TL and C, 2022). Effective management strategies are essential for patients experiencing hypertension during BTKi treatment. Common antihypertensive medications include ACEI, ARB, CCB, and beta-blockers (Samples et al., 2024). For high-risk patients, combination therapy may be necessary to manage blood pressure effectively. Furthermore, for those who require treatment discontinuation due to hypertension, reassessing the antihypertensive approach, adjusting medication doses, or temporarily suspending treatment may be necessary (K Sofija and F Michael, 2025).

Considering the patient’s underlying cardiovascular status, pre-treatment blood pressure, type of cancer, and potential BTKi side effects, blood pressure management should be tailored to the individual (L Ar TL and C, 2022). Before initiating BTKi therapy, assessing the patient’s cardiovascular risk (including a history of hypertension, atherosclerosis, etc.) and monitoring blood pressure is essential (Cohen et al., 2019a). Regular blood pressure monitoring during treatment is vital to facilitate timely adjustments to the treatment plan and prevent serious hypertension-related complications (LE Butel-Simoes et al., 2023). Recognizing the clinical significance of hypertension during BTKi treatment helps improve patient prognosis and reduce cardiovascular complications (Awan et al., 2022). Early hypertension detection and proper management safeguard treatment safety while optimizing anticancer efficacy. Therefore, cardiovascular risk assessment and regular blood pressure monitoring should be considered essential parts of BTKi therapy.

## 9 Future directions

### 9.1 Recommendations for future research and treatment strategies

Future research should further investigate the molecular mechanisms of BTKi-induced hypertension, with a particular emphasis on oxidative stress, endothelial dysfunction, and the regulation of vascular contraction. Although current studies have highlighted the correlation between BTKi and hypertension, the precise pathophysiological processes are still unclear, especially concerning the interaction and complexity of cellular signaling pathways. Future work could involve more detailed cellular models and animal studies to explore how BTKi affects blood pressure regulation by modulating various signaling pathways (such as PI3K/Akt, MAPK, NF-κB, Notch, and RhoA/ROCK), providing a theoretical foundation for the development of targeted therapeutic strategies.

The clinical management of hypertension during BTKi treatment should be further individualized, taking into account the patient’s baseline cardiovascular status, cancer type, and drug history. A comprehensive cardiovascular risk assessment should guide the selection of appropriate antihypertensive medications and the development of combination treatment strategies. Although current antihypertensive drugs such as ACE inhibitors (ACEI), angiotensin receptor blockers (ARB), and beta-blockers can control BTKi-induced hypertension to some degree, the efficacy and safety of these medications still need to be further validated through large-scale clinical trials. Future clinical research should focus on the effectiveness of various antihypertensive drug combinations, particularly in high-risk patients, and investigate novel cardiovascular protective therapies, such as antioxidants and endothelial function protectors, to alleviate the cardiovascular adverse effects of BTKi treatment.

Additionally, research in genomics and pharmacokinetics should continue to reveal the differences in patient responses to BTKi therapy, especially regarding individual variations in blood pressure response. Integrated with genomic data, Large-scale, multi-center clinical cohort studies could identify susceptibility genes for BTKi-induced hypertension, contributing to more precise treatment plans and improving safety and effectiveness.

In conclusion, as our knowledge of BTKi therapy advances, future research should focus on uncovering the molecular mechanisms and clinical features of BTKi-induced hypertension, identifying new therapeutic targets and management strategies, and facilitating more individualized and precise treatment of hypertension. This approach will reduce cardiovascular risks during BTKi therapy and optimize patient treatment outcomes.
